# Thermal equation of state of rhodium characterized by XRD in a resistively heated diamond anvil cell

**DOI:** 10.1038/s41598-024-78006-0

**Published:** 2024-11-04

**Authors:** Jose Luis Rodrigo-Ramon, Simone Anzellini, Claudio Cazorla, Pablo Botella, Aser Garcia-Beamud, Josu Sanchez-Martin, Gaston Garbarino, Angelika D. Rosa, Samuel Gallego-Parra, Daniel Errandonea

**Affiliations:** 1 Department of Applied Physics - Institute of Materials Science, Matter at High Pressure (MALTA) Consolider Team, University of Valencia, C/Dr. Moliner 50, Burjassot, 46100 Valencia Spain; 2 Department of Physics, Universitat Politècnica de Catalunya, Campus Nord B4-B5, Barcelona, 08034 Spain; 3 European Synchrotron Radiation Facility, 71 Avenue des Martyrs, Grenoble, CS 40220 38043 France

**Keywords:** Condensed-matter physics, Phase transitions and critical phenomena, Structure of solids and liquids

## Abstract

The high-pressure and high-temperature structural, mechanical, and dinamical stability of rhodium has been investigated via synchrotron X-ray diffraction using a resistively heated diamond anvil cell and density functional theory. The isothermal compression data have been fitted with a Rydberg-Vinet equation of state (EoS) with best-fitting parameters $V_0$ =55.046(16) Å$^3$, $K_0$ = 251(3) GPa, and $K'_0$ = 5.7(2). The thermal equation of state has been determined based upon the data collected following four different isotherms and has been fitted to a Holland and Powell thermal equation-of-state model with $\alpha _0=$3.36(7)x10$^{-5}$K$^{-1}$. The measured equation of state and structural parameters have been compared to the results of *ab initio* simulations. The agreement between theory and experiments is generally quite good. The present results solve controversies between previous studies which reported values of the bulk modulus from 240 to 300 GPa.

## Introduction

Transition metals have attracted significant interest owing to their distinctive electronic configuration arising from their partially filled *d* shell of electrons. Numerous pressure-induced phenomena have been documented, including topological transitions of the Fermi surface^1,2^. These phenomena can exert a profound influence on structural, thermal, and transport properties. Rhodium (Rh) is a 4*d* transition metal that belongs to the platinum (Pt) group of the periodic table, along with ruthenium (Ru), palladium (Pd), osmium (Os) and iridium (Ir). Platinum group metals are vitally important for a range of end-use applications in consumer and commercial products, as well as industrial processes^3^. Consequently recent extensive research efforts have been directed towards mapping the phase behaviors of these metals under extreme pressure-temperature (*P*-*T*) conditions^4–7^. Rhodium exhibits only a single electron in its highest occupied atomic orbital, specifically [Kr] 4d$^8$5s$^1$ and showcases exceptional properties, including a high melting point, hardness, and corrosion resistance. It is useful as an electrical contact material, as an alloying agent for hardening and improving the corrosion resistance of Pt and Pd, and in numerous alloys as a chemical catalyst^3,8^. Thanks to its inertness against corrosion, high melting point, low electrical resistance, and structural stability under a high-pressure environment, rhodium is suitable for applications under extreme conditions. For example, it is used for the manufacture of elements of nuclear reactors and high-power laser systems that carry intense thermal loads^9^. Such components work under a high energy concentration which create extreme conditions. For these reasons, several teams have started performing different studies on pure Rh at extreme *P*-*T* conditions, using various techniques.

At ambient conditions, Rh presents a face centred cubic (*fcc*) structure, that was shown to be stable up to at least 200 GPa at ambient *T* via a single-shock experiment^10^. Such a phase stability at ambient *T* was also confirmed up to 150 GPa by X-ray diffraction experiments (XRD) performed with diamond anvil cells (DAC) and using either Ne as pressure transmitting medium (PTM)^11–13^ or Bi^14^ as an alternative to He, as recently proposed by Storm et al.^15^. Theoretical studies have also suggested its structural stability up to 500 GPa^16–20^ and 1 TPa^21^. Concerning its $P-T$ phase diagram, a recent laser-heating DAC (LH-DAC) combined with XRD experiment performed by McHardy et al.^14^ confirmed the stability of the *fcc* phase of Rh up to at least 2700 K and 191 GPa. During this experiment, the authors also managed to extrapolate a thermal equation-of-state (EoS) for Rh, to use it as possible laser-heating coupler/standard for experiment performed at the European XFEL. Concerning its melting curve, the only available experimental data were collected in 1959 up to 10 GPa, using either *T* or the resistance evolution of a Rh wire resistively-heated loaded in a DAC^22^. On the other hand, while the available theoretical calculations performed with either *ab initio* simulations or molecular dynamics, are confirming the stability of the *fcc* phase of Rh up to 1 TPa and 16000 K, the melting curve predicted with the different technique are strongly diverging, with difference of the order of 2000 K already at 20 GPa^21,23,24^.

Previous experimental and theoretical works exhibit large discrepancies in the compression curve of Rh obtained at ambient *T*. Considering for example the three experiments performed in DAC using similar setups (Ne as PTM), the obtained bulk modulus and corresponding pressure derivatives vary from a minimum of 241 GPa and 5.31^13^ to 301 GPa and 3.1^12^. Finally, the data obtained by McHardy et al.^14^ using Bi as PTM, provide a bulk modulus and a pressure derivative of it that are somewhere in between the two values mentioned above. However, the use of Bi-V as PTM, instead of He or Ne, represents a technical novelty hence it needs to be further tested and validated. In addition, the only available thermal EoS of Rh was obtained from LH-DAC experiments^14^, which entails two main drawbacks related to the nature of the LH-DAC technique^25^: i) The minimum (high) *T* that is used to derive the thermal EoS is above 1000 K and, ii) the thermal gradients developed inside the sample during LH-DAC experiments, makes a precise characterization of thermal EoS quite complicated.

Because of the above described reasons, we decided to perform an additional characterization of the *P*-*T* behavior of Rh. In particular, to determine the isothermal equation of state at ambient *T*, combining XRD collected in DAC, with He as PTM, and first-principles density functional theory (DFT) calculations. We also explored the effect of pressure on the elastic constants via DFT calculations. Furthermore, we decided to expand our knowledge of Rh phase diagram between ambient conditions and, up to 50 GPa and 800 K, respectively, using XRD collected from resistively heated DAC (RH-DAC). This guaranteed a homogeneous and better controllable heating of the entire sample, facilitating the characterization of its thermal EoS and reducing the experimental uncertainties.^26^

## Results

### Evolution at ambient T

**Fig. 1:**
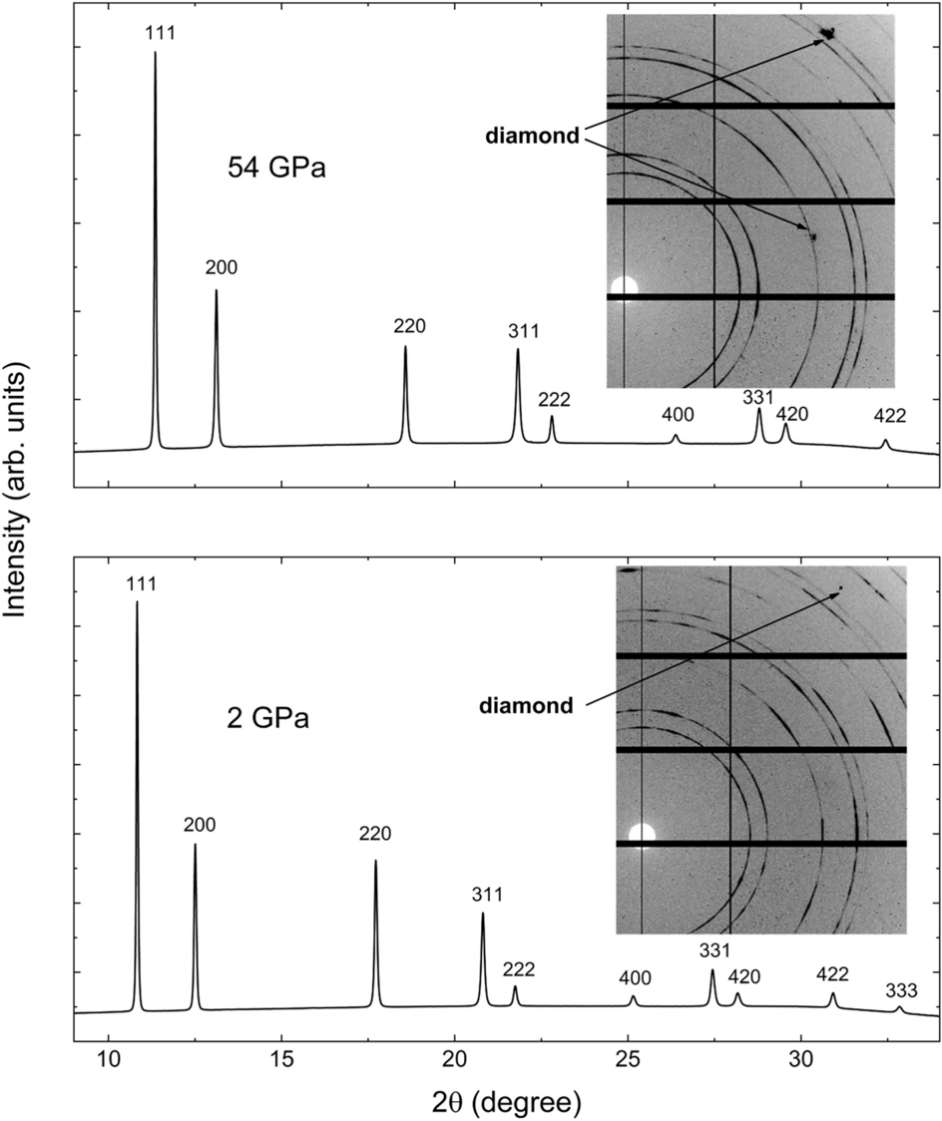
Integrated powder X-ray diffraction patterns of Rh at low (bottom) and the highest (top) *P* reached in the present experiment. The insets show a part of the recorded raw diffraction images. The diamond contribution to the X-ray raw patterns are masked during the integration.

One of the experiments was performed at ambient *T* under quasi-hydrostatic conditions, using He as PTM and following the procedure described in the Method section. During this experiment, *P* was increased from ambient up to 54.5 GPa with *P* increments below 2 GPa. In agreement with previously reported works^11–14^, Rh maintained its *fcc* structure in all the investigated *P* range. Figure 1 shows the image plates and the corresponding integrated XRD patterns obtained for Rh at low and the highest *P* reached in the present experiment. From the reported data, it is possible to observe how the texture of the nine diffraction rings of Rh (111, 200, 220, 311, 222, 400, 331, 420 and 422) remains the same (highly oriented powder) in the investigated range. However, a slight broadening of the measured peaks is also observed. From a qualitative analysis of the hydrostatic conditions of the sample^7,27^ shown in Table 1, it is possible to observe how at the highest *P* reached in the present experiment, the difference between the measured *d*-spacing and the theoretical (hydrostatic) one are below 0.2 $\%$. As such a deviation corresponds to the uncertainties on the measurement of each diffraction peak,^7,27–29^ we can conclude that macroscopic non-hydrostatic effects are below the detection limits of the present experiments.

**Table 1:** Measured reflections for rhodium and gold at 54 GPa. (*hkl*) are the Miller indices of the reflection. $d_m$ is the corresponding interreticular distance measured by individual peak fitting. $d_c$ is the interreticular distance calculated by a fit of the whole spectrum.

	Rh			Au		
	$\mathbf {d_m}$(Å)	$\mathbf {d_c}$(Å)	$\mathbf {|d_m-d_c|}$(Å)	$\mathbf {d_m}$(Å)	$\mathbf {d_c}$(Å)	$\mathbf {|d_m-d_c|}$(Å)
(111)	2.0889	2.0888	0.0001	2.2072	2.2072	0.0000
(200)	1.8095	1.8090	0.0005	1.9115	1.9107	0.0008
(220)	1.2800	1.2792	0.0008	1.3516	1.3515	0.0001
(311)	1.0917	1.0909	0.0008	1.1526	1.1518	0.0008
(222)	1.0452	1.0444	0.0008	1.1036	1.1031	0.0005
(400)	0.9056	0.9045	0.0011	0.9557	0.9545	0.0012
(331)	0.8310	0.8300	0.0010	0.8770	0.8765	0.0005
(420)	0.8101	0.8090	0.0011	0.8548	0.8538	0.0010
(422)	0.7397	0.7385	0.0012	0.7803	0.7794	0.0009
(333/511)	0.6972	0.6963	0.0009	0.7357	0.7343	0.0014

**Table 2:** The unit-cell parameters of Rh and Au at ambient temperature as a function of pressure. All values are obtained using He as pressure transmitting medium. The pressures measured with the EoS of gold are all reported in GPa. The lattice parameters are reported in Å. Experimental uncertainty on lattice parameters is lower than 0.003 Å. Uncertainty on pressure measurement increases from 0.05 GPa at 1 GPa to 0.7 GPa at 54 GPa.

$\mathbf {a_{Rh}}$ (Å)	$\mathbf {V_{Rh}}$ (Å$^3$)	$\mathbf {a_{Au}}$ (Å)	$\mathbf {V_{Au}}$ (Å$^3$)	P (GPa)	$\mathbf {a_{Rh}}$ (Å)	$\mathbf {V_{Rh}}$ (Å$^3$)	$\mathbf {a_{Au}}$ (Å)	$\mathbf {V_{Au}}$ (Å$^3$)	P (GPa)
3.804	55.025	4.078	67.808	0.096	3.704	50.811	3.930	60.695	25.155
3.803	55.002	4.077	67.766	0.201	3.701	50.711	3.927	60.539	25.916
3.802	54.978	4.076	67.722	0.310	3.698	50.557	3.921	60.299	27.102
3.801	54.906	4.073	67.59	0.641	3.694	50.394	3.916	60.047	28.377
3.800	54.871	4.072	67.525	0.806	3.690	50.262	3.911	59.844	29.432
3.797	54.730	4.067	67.269	1.464	3.687	50.125	3.907	59.634	30.537
3.792	54.527	4.060	66.900	2.441	3.684	49.995	3.903	59.435	31.611
3.788	54.341	4.053	66.567	3.351	3.680	49.847	3.898	59.208	32.855
3.782	54.108	4.044	66.153	4.525	3.678	49.744	3.894	59.052	33.728
3.777	53.889	4.036	65.768	5.661	3.675	49.617	3.890	58.859	34.825
3.775	53.815	4.034	65.638	6.052	3.671	49.490	3.886	58.667	35.934
3.770	53.568	4.025	65.210	7.379	3.667	49.311	3.880	58.397	37.529
3.763	53.274	4.015	64.706	9.012	3.665	49.230	3.877	58.275	38.260
3.755	52.961	4.004	64.176	10.814	3.662	49.109	3.873	58.093	39.371
3.753	52.848	4.000	63.986	11.483	3.659	49.007	3.870	57.941	40.316
3.747	52.602	3.991	63.575	12.966	3.657	48.903	3.866	57.784	41.303
3.741	52.372	3.983	63.196	14.390	3.655	48.828	3.864	57.673	42.013
3.737	52.171	3.976	62.866	15.671	3.655	48.814	3.863	57.652	42.147
3.734	52.061	3.972	62.686	16.387	3.653	48.756	3.861	57.564	42.709
3.733	52.034	3.972	62.642	16.561	3.651	48.648	3.858	57.403	43.758
3.728	51.830	3.965	62.313	17.907	3.629	47.810	3.830	56.162	52.398
3.724	51.649	3.958	62.022	19.133	3.629	47.789	3.829	56.131	52.630
3.720	51.488	3.953	61.764	20.246	3.628	47.740	3.827	56.059	53.162
3.718	51.383	3.949	61.596	20.985	3.626	47.674	3.825	55.962	53.889
3.714	51.215	3.944	61.330	22.180	3.624	47.612	3.823	55.870	54.586
3.709	51.038	3.938	61.050	23.468

**Table 3:** EOS parameters of Rh measured and calculated in different experiments. The volume $V_0$, the bulk modulus $K_0$ and its pressure derivative $K'$ are listed. Experimental methods and EoS formulation are specified. Notice that the parameters used where obtained not always using the same equation of states. More information on the equations of states can be found in the corresponding references.For completeness, we have also included the references for the pressure scales used in each work.

Reference	$\mathbf {V_0}$ (Å$^3$)	$\mathbf {K_0}$ (GPa) $\mathbf {K'}$	PTM	Pressure gauge	EoS	Method	Pressure Range (GPa)
This study	55.046(16)	251(3), 5.7(2)	He	Au^28^	Vinet	AD-XRD DAC	0-55
This study	55.045(15)	252(3), 5.5(2)	He	Au^28^	BM3	AD-XRD DAC	0-55
^14^	55.056(8)	258(3), 5.36(9)	Bi	Bi^30^	AP2	AD-XRD DAC	0-180
^13^	55.062(63)	241.3(65), 5.34(24)	Ne	Tungsten^31^	Vinet	AD-XRD DAC	0-60
^12^	54.92(7)	301(9), 3.1(2)	Ne	Ruby^32^	BM3	AD-XRD DAC	0-65
^11^	54.952	270.0, 4.756	Ne	Cu	BM3	AD-XRD DAC	31-83
^10^	55.008(4)	269(8), 4.5(1)				Single-shock	0-212 GPa
^33^		264(1)				ultrasound	
^34^	54.83	299.4, 4.9				Theo	0-300 GPa
This study	53.8	299, 4.9			BM3	Theo-DFT	0-140

**Fig. 2:**
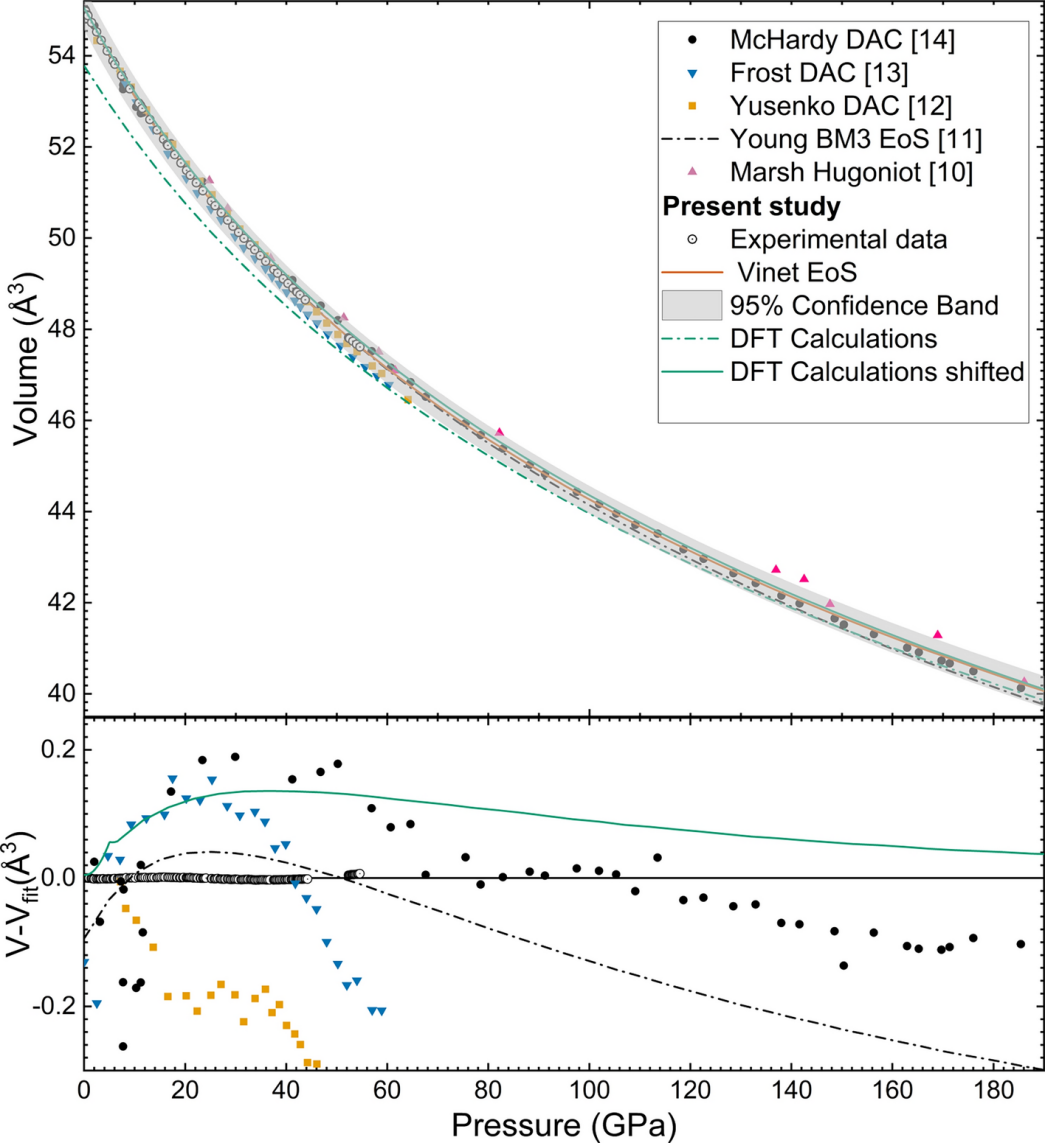
(Top) Measured and calculated volume of rhodium as a function of pressure compared with literature data and *ab initio* calculations. (Bottom) Difference between measured and fitted volume, Vinet formulation with $V_0$=55.046(10), $K_0=$ 251(3), $K'$= 5.7(2).

**Fig. 3:**
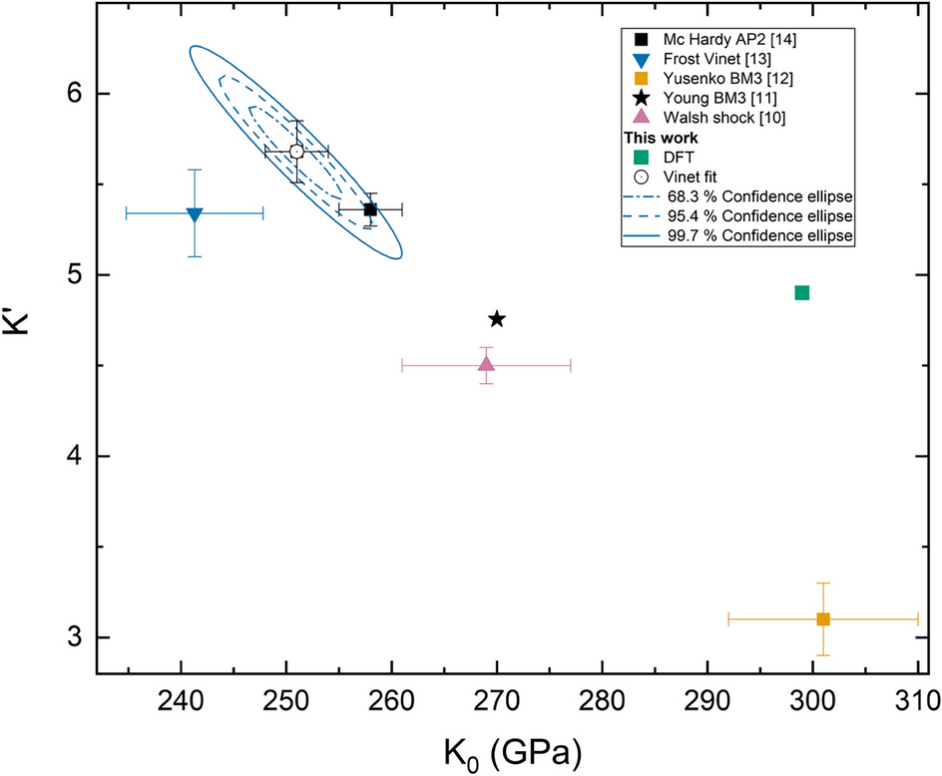
$K'$ versus $K_0$ values as obtained from different studies. Confidence ellipses from the present experiments are shown.

The obtained results for Rh and Au, as well as the corresponding *P* are reported in Table 2. Whereas, the corresponding compression curve is represented in Fig. 2, together with previous results and the present *ab initio* calculations. The corresponding bulk modulus $K_0$, its pressure derivative $K'_0$ and the volume $V_0$ at ambient temperature have been determined from a least-square fit of the entire set of data to a Rydberg-Vinet^35^ and a third-order Birch-Murnaghan (BM3) equation of state (EoS) using the EOSFit7c sofware^36^. The obtained values are reported in Table 3 together with the current and literature EoS parameters.

As shown in Fig. 2, the compression curve obtained in this study is in excellent agreement, up to the highest pressure probed, with the one reported by Young *et. al*^11^ who used Ne as PTM. However, at higher *P* the divergence between the two EoS becomes more pronounced. In particular, the differences in the volumetric compression between the two reported EoS are less than 0.2$\%$ and $1\%$ up to 60 GPa and 200 GPa, respectively (if we extrapolate our data). Comparing the values in Table 3, they are reporting a $V_0$ slightly smaller than ours ($\sim$ 0.2$\%$) and a larger and smaller values for $K_0$ and $K'_0$, respectively. It should be noted that there is a good agreement also with the recently published EoS by Mc Hardy *et. al*^14^ with differences in the volume compression of $\sim$ 0.2$\%$ up to 60 GPa and smaller than 0.6 $\%$ when extrapolating our data up to 200 GPa. Differences between the experimentally measured volume by McHardy *et. al* and the fitted volume according to our Vinet EoS model are less than 0.25 Å$^3$. We can therefore conclude that, in the investigated *P*-range, compression data obtained using He as PTM are indeed consistent with the ones obtained with Bi as PTM. On the other hand, the present results differ from the rest of DAC experiments performed with Ne as PTM. In particular, we are reporting a considerably higher compressibility than Yusenko *et. al*^12^ (smaller bulk modulus). On the other hand, Frost *et. al*^13^ reported a bulk modulus $\sim 4\%$ smaller than ours. Also should be noted the agreement between our EoS and the one obtained with single-shock on the principle Hugoniot up to a maximum pressure of 212 GPa by Walsh et. al^10^. In Fig. 3 we are also reporting the values of $K'_0$ as a function of $K_0$ as reported in the various studies. Discrepancies in compression data between different works could be related to differences in the pressure gauge. This is a fact that should be further studied in future studies, the different pressure scales used in each work can be found in Table 3.

The theoretical compression curve obtained in the present study with first-principle DFT is also reported in Fig. 2. Our calculations underestimate $V_0$ since they are computed at 0 K. Therefore, we corrected the theoretical EoS to compare them with the experimental data and both DFT curves are represented in Fig. 2 with dashed (original) and continuous (corrected) green lines. Concerning the results, the present calculations give a slightly smaller compressibility than the experiments (larger bulk modulus). However, the difference between the experimentally measured bulk modulus and the one obtained via *ab initio* calculations is less than 20 $\%$. Beyond 30 GPa , calculations and experiment provide very similar volumetric compressions, with the two EoS running nearly parallel to each other, as a consequence of the slightly smaller calculated $K'_0$.

**Fig. 4:**
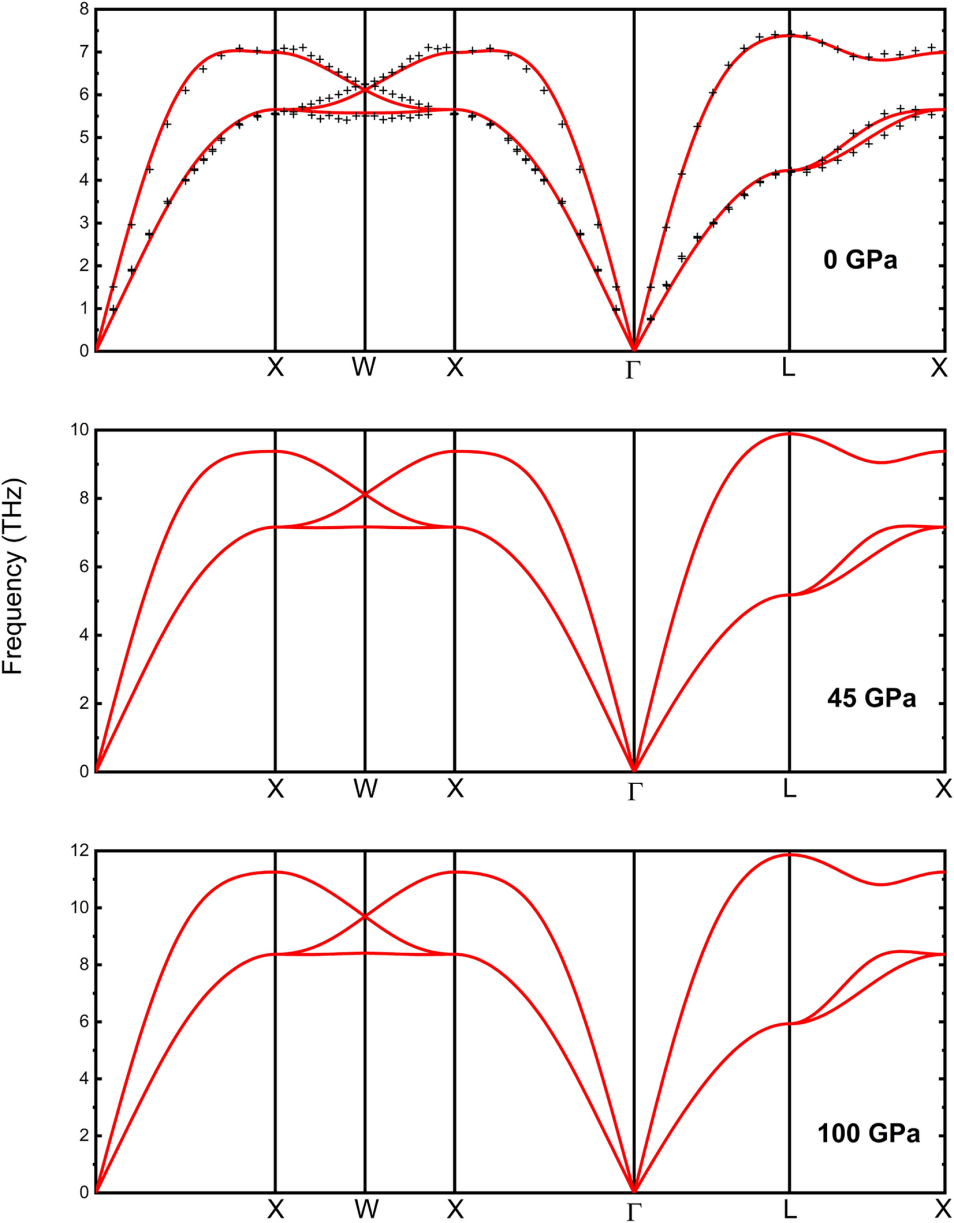
Simulated phononic dispersion curves of *fcc* Rh at 0 GPa, 45 GPa and 100 GPa (red solid line). Experimental values from neutron scattering obtained by Eichler *et. al*^37^ (black dots) at ambient pressure are also represented.

The phonon dispersion obtained via *ab initio* calculations at 0 GPa is shown in Fig 4 together with experimental values^37^. The dispersion at ambient pressure agrees perfectly with that reported by Eichler et al.^37^ obtained from neutron scattering experiments. Since the simulation is consistent with the experimental results at 0 GPa we have also studied the phonon dispersion at 45 and 100 GPa. The results are represented in Fig. 4. The dispersion of phonons does not show substantial changes in the *P*-range reached in the study. The main change is the increase of the slope of the two phonon branches that are linear near the $\Gamma$ point of the Brillouin zone, which indicates that the sound speed increases with the density of Rh under compression. We have also calculated the elastic constants. The values of the elastic constants at ambient *P* obtained by our calculations are shown in Table 4 together with experimental values^38,39^.The reported values from DFT calculations show quite good agreement with the experimental values, the differences among the two amounting to less than $10 \%$.

**Table 4:** DFT calculated elastic constants and elastic moduli of Rh at 0 GPa. Experimental values of the elastic constants at 0 GPa from references^38,39^ have been added for comparison.

	**Elastic moduli (GPa)**	This work	**Elastic constants (GPa)**				
Bulk modulus	$B=\frac{C_{11}+2C_{12}}{3}$	293.7		This work DFT	Exp.^38^	Exp.^39^	Exp.^33^
Shear modulus	$G=\frac{1}{2}\left( \frac{C_{11}+2C_{12}+3C_{44}}{5}+\frac{5C_{44}(C_{11}-C_{12})}{4C_{44}+3(C_{11}-C_{12})}\right)$	172.3	$C_{11}$	466.894	422.1	416.5	418.9(25)
Poisson’s ratio	$\nu = \frac{3B-2G}{3(3B+G)}$	0.169	$C_{12}$	207.13	191.9	197.5	188.6(12)
Young modulus	$E=\frac{9BG}{3B+G}$	432.4	$C_{44}$	208.217	194	184	195.1(5)

**Fig. 5:**
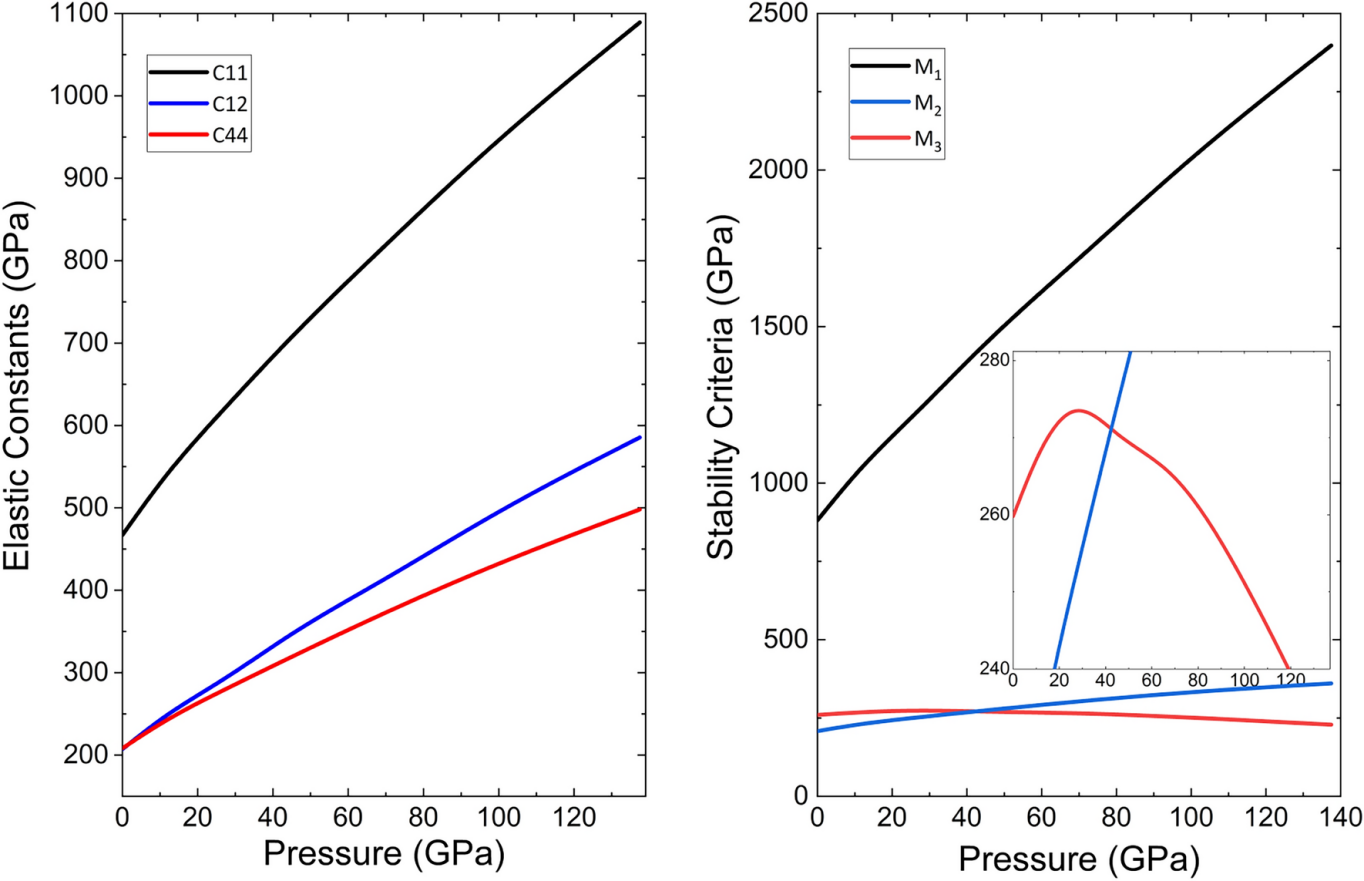
(Left) Evolution of the elastic constants $C_{ij}$ of rhodium with pressure obtained from *ab inito* calculations. (Right) Generalized Born stability criteria ($\hbox {M}_{i}$) versus pressure for Rh. The inset shows a magnified region of the plot where it can be observed the pressure at which $M_2$ starts to decrease.

Figure 5 shows the evolution of the elastic constants with pressure for Rhodium obtained from our calculations. We have checked also the mechanical stability of the crystal with the elastic constants. For cubic crystals mechanical stability under *P* occurs under the following generalised Born criteria^40,41^:

$$\begin{aligned} M1= & C_{11}+2C_{12}+P>0 \\ M2= & C_{44}-P>0 \\ M3= & C_{11}-C_{12}-2P>0 \end{aligned}$$

These conditions and their evolution with pressure are represented in Fig. 5. Our results show that *fcc* Rh is dynamically stable up to the maximum *P* reached with our calculations (140 GPa). However, as can be seen from the inset in Fig. 5$\hbox {M}_{2}$ starts to decrease at 30 GPa. A linear extrapolation of the trend of $M_{2}$ would lead to $\hbox {M}_{2}$ < 0 at around 350 GPa, violating one of the generalized Born criteria of stability. Previous theoretical studies suggest the stability of the *fcc* phase up to 500 GPa^16–20^ and 1 TPa^21^, confirming that no phase transition takes place in Rh up to extreme pressures. Notice that failure through $\hbox {M}_{2}$ < 0 is characterized by symmetry breaking with coupling of shear modes under volume conservation. This suggests that a face-centred tetragonal (*fct*) structure, also known as $\hbox {A}_6$, a distortion of the *fcc* structure and the mono-atomic analog of the $\hbox {L}_{10}$ structure, could both be potential candidates as HP phase of Rh. This structure has exactly the same symmetry as the body-centred tetragonal $\hbox {A}_6$ structure. Therefore it could give a Bain path for a transformation of *fcc* into *bcc* at extremely high-pressure. In the $\hbox {A}_6$ structure, c/a is near the *fcc* ratio of $\sqrt{2}$, while in the $\hbox {A}_6$ structure, c/a is near the *bcc* ratio of 1.

The bulk modulus can also be obtained from the elastic constants. For a cubic crystal it can be expressed in the form^42^:

$$\begin{aligned} B=\frac{C_{11}+2C_{12}}{3} \end{aligned}$$

The obtained bulk modulus differs by less than 20$\%$ with our experimental data. We also report the isotropic shear modulus *G*, elastic moduli *E*, and the Poisson’s ratio $\nu$. These parameters describe the major elasticity properties for a material. In our case they are defined by the equations described in^42^ in which the shear modulus is computed from the average of the Voigt and Reuss bounds^43^. The obtained values are included in Table 4. The value of the shear modulus shows that in Rh shear stiffnes is smaller than compressional stiffnes. In addition, the large value of the Young modulus shows that the tensile or compressive stiffness when the force is applied lengthwise is much larger than the resistance of Rh to hydrostatic volumetric changes. Finally the value of the Poisson ratio is much smaller than that of Cu and similar to that of cast iron indicating that Rh is a brittle material.

## High Temperature high pressure evolution

**Fig. 6:**
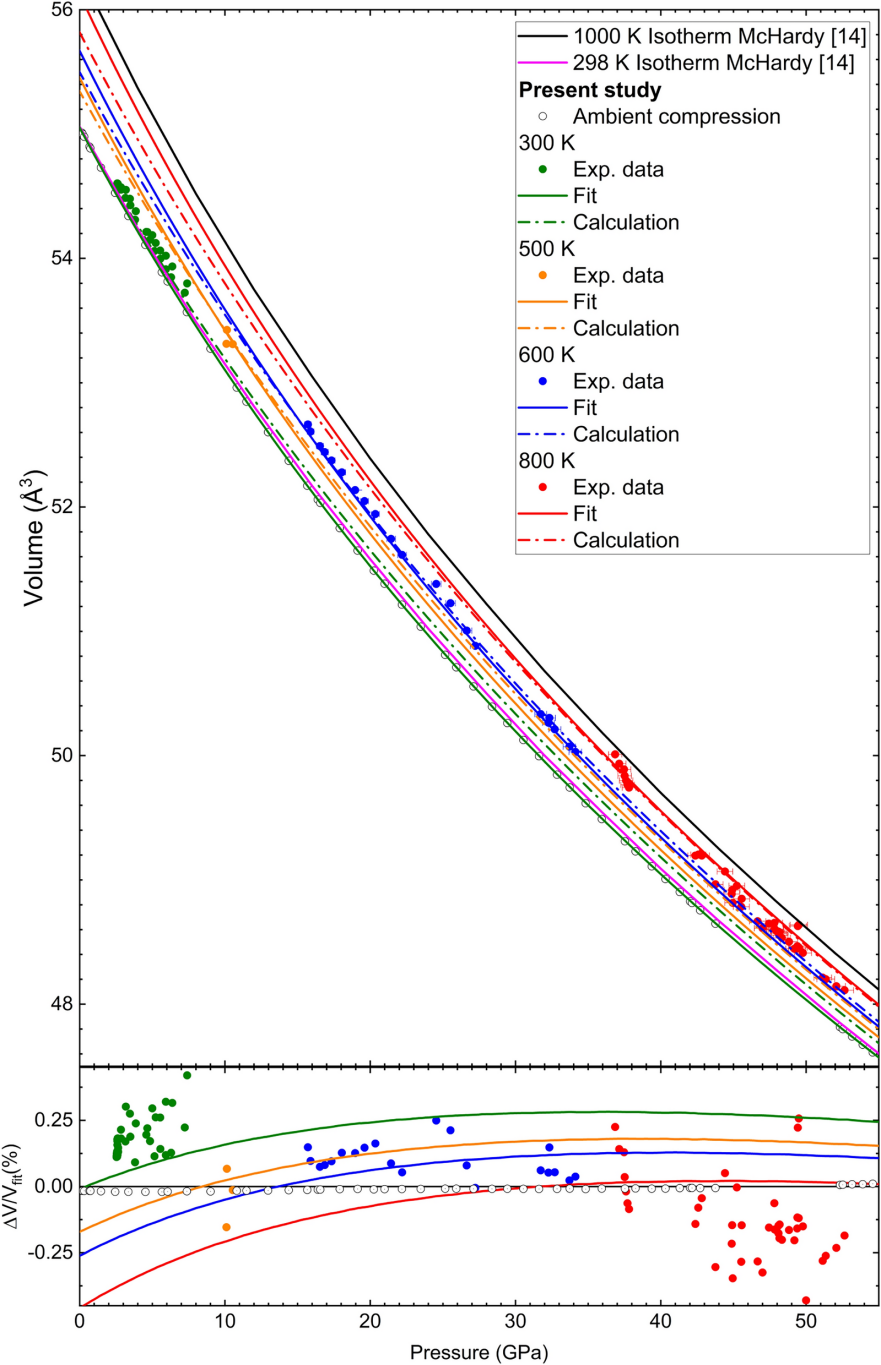
(Top) Compression curves of Rh obtained at different temperatures. The symbols represent different temperature groups whereas the lines represent the corresponding *P*-*V*-*T* EoS fit. The same color code has been used for experiments and fits. The dashed lines represent the isotherms obtained by *ab initio* theoretical calculations where the underestimation of $V_0$ has been corrected. Two isotherms (298 and 1000 K) from a previous experiment^14^ are also represented. (Bottom) Difference between the experimental, DFT calculations and fitted volume $\left( \frac{V-V_{fit}}{V_{fit}}(\%) \right)$ with Holland and Powell formulation with $\alpha _0=3.36(7)$x$10^{-5} K^{-1}$.

XRD experiments performed up to 50 GPa and 800 K have shown that Rh remains in the *fcc* structure with no evidence of phase transitions or structural distortions. The pressure and temperature dependence of the Rh unit-cell obtained from four isotherms together with the RT results are shown in Fig. 6. Also results from previous high- and room-temperature studies are included^14^. The results from the *HT* experiments carried out using KCl as PTM and pressure gauge^44^ are consistent with both the one obtained at room *T* using either He or Bi as PTM^14^.

Starting from the presented data it was possible to establish the $P-V-T$ equation of state (EoS) for *fcc*-Rh using the EOSfit software suite^36^. According to the formalism described in Angel et al.^36^ *P* at *HT* can be described as the sum of *P* at a reference *T* condition (typically 300 K) and the thermal *P* with increasing *T* evaluated along isochors, and hence it can be expressed as:

$$\begin{aligned} P(V,T)=P(V,300 K)+P_{Th}(T) \end{aligned}$$

Where the *P*(*V*, 300*K*) is the isothermal EoS of the material at ambient *T* and $P_{Th}(T)$ the thermal pressure. In the present work *P*(300*K*) was modelled with a third-order Vinet EoS, whereas the thermal part was modeled following Holland and Powell:

$$\begin{aligned} P_{Th}(T)=\alpha _0 K_0 \frac{\Theta _E}{\zeta _0}\left( \frac{1}{exp(\Theta _E/T)-1}-\frac{1}{exp(\Theta _E/300)-1} \right) \end{aligned}$$

Where $\Theta _E$ is the Einstein *T* of the material (350 K for Rh), $K_0$ is the bulk modulus obtained from the isothermal EoS at ambient *T*, $\alpha _0$ is the thermal expansion coefficient at 300 K and $\zeta _0$ is expressed as:

$$\begin{aligned} \zeta _0=\frac{(\Theta _E/300)^2 exp(\Theta _E/300)}{(exp(\Theta _E/300)-1)^2} \end{aligned}$$

This simple two-parameter thermal pressure model captures both the ambient pressure isobaric expansion behavior and the resistive heating data. As shown in Fig. 6, data grouped considering T ranges of 100 K are well described by a 3rd order Vinet EoS and a thermal pressure modeled according to Holland and Powell expression with $K_0$=251(3) GPa, $K'_0$=5.7(2), $V_0$=55.046(16) and Å$^3$ $\alpha _0=3.36(7)$x$10^{-5} K^{-1}$. In the fit of the *P*-*V*-*T* EoS the above given values for $K_0$, $K'_0$ and $V_0$ have been considered as fixed.

In Fig. 6, the isotherms obtained from the present DFT calculations at 300, 500, 600 and 800 K are presented and compared to the ones obtained from the *P*-*V*-*T* EoS fit of the experimental data. As we have mentioned in the previous section, our DFT calculations underestimate $V_0$. For comparison reasons the underestimation in $V_0$ extracted from the ambient compression data has been corrected for the four isotherms computed. As can be seen from Fig. 6 the simulated isotherms agree perfectly with the experimental data obtained. For low values of *P* and higher *T* the *ab initio* simulated isotherms give a bigger underestimation of the volume compared with the isotherms obtained for our *P*-*V*-*T* EoS. Regarding the volumetric evolution with pressure beyond 15-20 GPa and for temperatures up to 800 K, calculations and experimental isotherms provide very similar volumetric compressions, with isotherms running nearly coincident or parallel for the range of P-T studied. Differences between the computed value of volume with the fitted *P*-*V*-*T* EoS and the simulated volume of the *ab initio* calculations are smaller than 0.25 Å$^3$ for pressures up to 60 GPa.

**Fig. 7:**
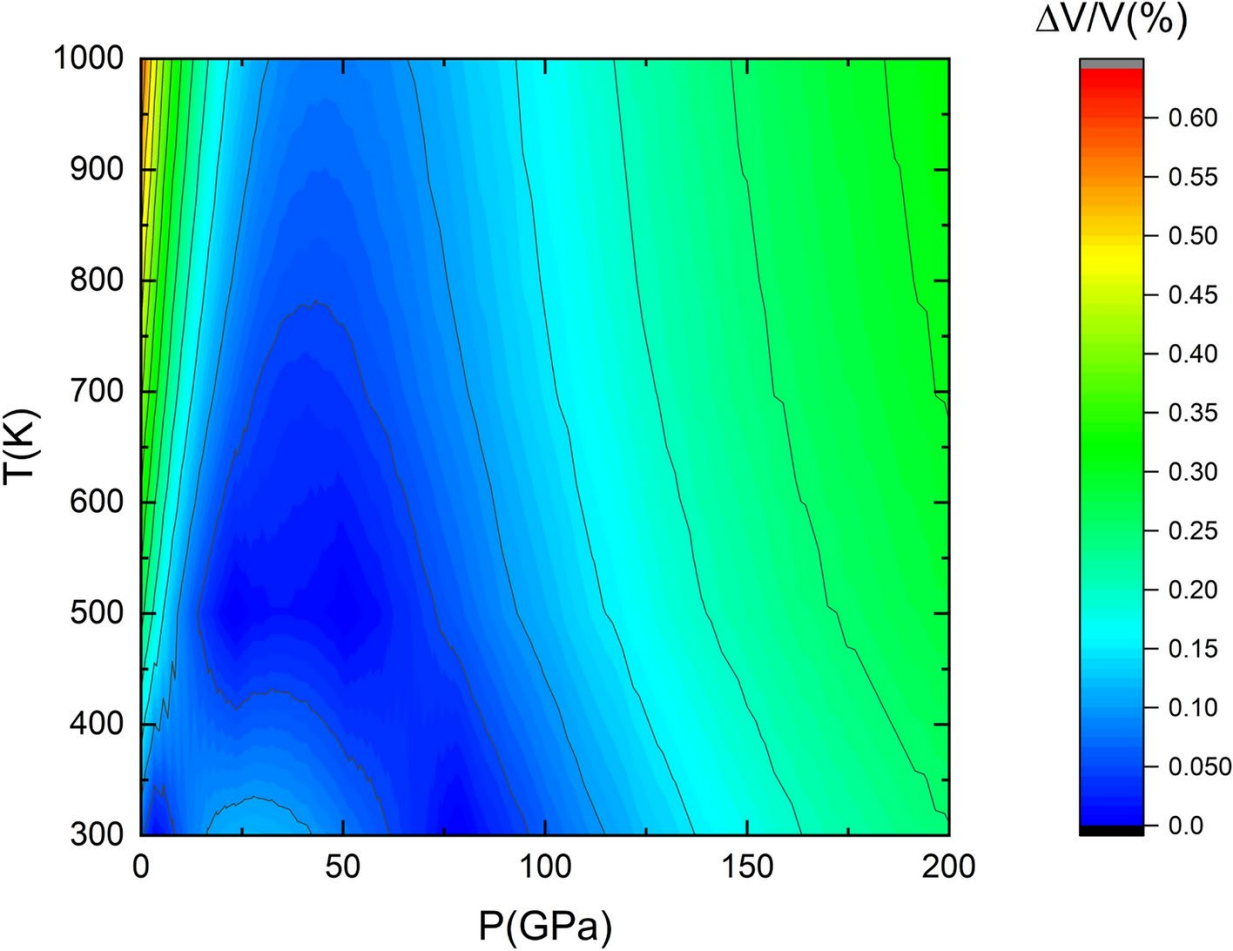
2D pressure-temperature (P-T) map displaying the differences in computed volume between the thermal equation of state (EoS) by McHardy et al.^14^ and the EoS derived in this work. The map highlights the variations in volumetric predictions across the P-T space.

Moreover the obtained isotherms for the present study are consistent with the recently published results by McHardy *et. al*^14^ for LH-DAC. In Fig. 6 two isotherms at 298 K and 1000 K from the previous work have been represented. It can be seen that there is a good agreement between the fitted isotherm at 300 K of this workand that reported by McHardy *et. al*^14^ (green and pink solid lines in Fig. 6).The differences in the calculated volume between both isotherms in the pressure range 0-60 GPa are less than 0.1$\%$. Regarding the parameter $\alpha _0$ they obtained with the same Holland Powell model a value $\alpha _0=2.050(12)$x$10^{-5} K^{-1}$ for their AP2 EoS. Our best fit obtained a value of $\alpha _0=3.36(7)$x$10^{-5} K^{-1}$ for the Vinet EoS. The tension between the two values of $\alpha _0$ could be related to the different temperature range covered by studies and the different EOS model used in both studies. A two-dimensional pressure-temperature (P-T) map was generated to visualize the differences in the computed volume between the thermal equation of state (EoS) derived by McHardy et al.^14^ and the one obtained in the current study. The map shown in figure 7 highlights the consistency in volumetric predictions across a range of pressures and temperatures, providing a comparative assessment of the models’ behavior under varying thermodynamic conditions In the pressure and temperature range considered in this study (0-60 GPa and 300-900 K), the discrepancies in the calculated volumes between the two equations of state (EoS) remain below 0.4 %, highlighting the high degree of consistency between the two models. Our findings aligned with the pevious experimental results provide an accurate P-V-T EoS that allows the use of Rh as a P-T gauge for future experiments.

## Conclusions

In this work, the volumetric compression of Rh at ambient *T* has been investigated experimentally under quasi-hydrostatic conditions using a combination of synchrotron XRD and DAC with He as PTM and theoretically, using DFT calculations. The results provide an EoS in good agreement with the EoS obtained using Bi as PTM, confirming the hypothesis of Storm *et al.*^15^ of Bi being a PTM as hydrostatic as He, at least up to 50 GPa. A fit of the present compression curve with a Vinet EoS provides $V_0$ =55.046(16) Å$^3$, $K_0$ = 251(3) GPa, and $K'_0$ = 5.7(2).

From the DFT calculations it was possible to obtain the elastic constants and elastic moduli of Rh and its phonon dispersion as a function of *P*. The obtained vibrational modes confirm *fcc* Rh to be structurally and dynamically stable up to at least 140 GPa. However, an extrapolation of the obtained elastic constants to higher *P* leads to a value of $\hbox {C}_{44}$-*P* that becomes lower than 0 at 350 GPa. Such a violation of the generalized Born criteria suggests the possible presence of a phase transition of Rh at this *P*. We are proposing an $\hbox {A}_6$ structure as the potential new *HP* phase of Rh above 350 GPa. Finally, from synchrotron XRD data collected using RH-DAC in a *P*-*T* range between ambient and 50 GPa and 800 K, respectively, we extracted a thermal EoS of Rh that follows the Holland and Powell model with $\alpha _0=$3.36(7)x10$^{-5}$K$^{-1}$. The extrapolated thermal EoS, reproduces well also ther results obtained by McHardy *et al.*^14^ from LH-DAC experiment and, are in good agreement with the one obtained from the present *ab initio* calculation once taken into account of the *P*-shift due to the 0 K nature of the calculations itself.

## Methods

### Experimental

Two membrane DACs equipped with bevelled diamonds of 150x300 $\mu$m and 250 um $\mu$m culets were used for the cold compression curve and the *HP*-*HT* one, respectively. In both cases, Re foils were used as gasket materials: pre-indented and laser-drilled to obtain a high pressure chamber for the sample. For the ambient *T* compression experiment, the sample loading was performed to optimize hydrostaticity. In particular, a grain of Rh (Good Fellow 99.9$\%$) was loaded at the centre of the diamond, together with a grain of Au and a couple of ruby sphere placed few $\mu$m away from the Rh sample. The high pressure chamber was then filled with He. The presence of He in the DAC was confirmed via ruby fluorescence method^45^ *i.e.* by measuring the presence of pressure in the high pressure chamber. For the HP-HT compression, special internal heaters formed by amorphous boron composite gaskets^46^ were prepared. The Rh sample (same powder as for the cold compression) was embedded in KCl, acting as both PTM and *P* standard. The experiments were performed at the extreme conditions beamline ID15B^47^ of the ESRF (European Synchrotron Radiation Facility), were the X-rays were tuned to 30 keV and focussed down to 5x5 $\mu$m$^2$. XRD signals were collected on the beamline’s large area EIGER2 X 9M CdTe (340x370 mm) flat panel detector. The sample-to-detector distance (261.7599 mm) was obtained from the XRD signal of a Si standard, following standard procedure and using the calibration routine of the DIOPTAS sofware suite^48^. Thanks to the micrometric nature and the high brilliance of the used X-ray beam, during the cold compression experiment, two XRD patterns were collected at each *P* step, one entirely from the Rh sample and one from the Au calibrant at 1 s time interval. The actual *P* in the sample chamber was extracted from the corresponding volumetric compression of Au, following the calibration of Takemura and Dewaele^28^. The *HP*-*HT* compression/decompression ramps were performed using the internal resistive-heaters developed at the ESRF^46^. The currents passing through the heaters mounted on each seat of the cell were regulated using an eurotherm controller connected to a power supply. The temperatures at the culet and on the back plates of each diamond were monitored using K-type thermocouples, with each diamond having its own K-type thermocouple. In this case, the *P* inside the cell was measured from the thermal equation of state of KCl-B2 as reported in Dewaele *et al.*^44^ under the hipothesis that both the KCl and the Rh were experiencing the same *T*. Such an assumption is quite reliable considering the nature of the internal RH-DAC experiment.^25,26^ In both cases, mask were applied on a per image basis and the XRD pattern were azimuthally integrated in DIOPTAS. A Pawley refinement was performed on each obtained integrated pattern with the TOPAS academic suite^49^, in order to extract the lattice parameters of both the sample and the adopted *P* calibrant. The obtained volumetric compression as a function of *P* (and *T*) were analysed with the EOSfit software suite^36^ so to extract the corresponding equation of states.

## Computational

First-principles calculations based on density functional theory (DFT) were performed to analyze the equation of state and structural and vibrational properties of fcc Rh under pressure. We performed these calculations with the VASP code^50^ by using projector augmented-wave method potentials^51^. The electronic states 4*d* and 5*s* were considered as valence. Wave functions were represented in a plane-wave basis truncated at 750 eV. By using these parameters and dense $\textbf{k}$-point grids of $16 \times 16 \times 16$ for integration within the Brillouin zone (BZ), energies were converged to within 0.5 meV per formula unit. In the geometry relaxations, a force tolerance of 0.005 eV$\cdot$Å$^{-1}$ was imposed. The semi-local DFT exchange-correlation functional GGA-PBEsol^52^ was employed in all the calculations. The elastic constants of fcc Rh and their dependence on pressure were obtained through the calculation of the stress-strain tensor at different volumes.

*Ab initio* phonon frequencies were calculated with the small-displacement method^53,54^. In this approach, the force-constant matrix is calculated in real-space by considering the proportionality between atomic displacements and forces. The quantities with respect to which our phonon calculations were converged include the size of the supercell, size of the atomic displacements, and numerical accuracy in the sampling of the BZ. We found the following settings to provide quasi-harmonic free energies converged to within 1 meV per formula unit: $4 \times 4 \times 4$ supercells (where the figures indicate the number of replicas of the unit cell along the corresponding lattice vectors), atomic displacements of 0.02 Å, and $\textbf{k}$-point grids of $4 \times 4 \times 4$. The value of the phonon frequencies were obtained with the PHONOPY code^55^. In using this code, we exploited the translational invariance of the system, imposing the three acoustic branches to be exactly zero at the center of the BZ, and applied central differences in the atomic forces. The theoretical equations of state at *P*–*T* conditions were calculated within the quasi-harmonic approximation^53,54^ by performing a total of ten phonon calculations at different volumes.

## Supplementary Information

Supplementary Information.

## Data Availability

The datasets generated during and/or analysed during the current study are available from the corresponding author on reasonable request.

## References

[1] Glazyrin, K. et al. Importance of correlation effects in hcp iron revealed by a pressure-induced electronic topological transition. *Phys. Rev. Lett.* **110**, 117206. 10.1103/PhysRevLett.110.117206 (2013). 25166573 10.1103/PhysRevLett.110.117206

[2] Dubrovinsky, L. et al. The most incompressible metal osmium at static pressures above 750 GPa. *Nature* **525**, 226–229. 10.1038/nature14681 (2015). 26302297 10.1038/nature14681

[3] Hughes, A. E., Haque, N., Northey, S. A. & Giddey, S. Platinum group metals: A review of resources, production and usage with a focus on catalysts. *Resources* **10**, 10.3390/resources10090093 (2021).

[4] Anzellini, S., Burakovsky, L., Turnbull, R., Bandiello, E. & Errandonea, D. P–V–T equation of state of iridium up to 80 GPa and 3100 K. *Crystals* **11**, 10.3390/cryst11040452 (2021).

[5] Baty, S. R., Burakovsky, L., Luscher, D. J., Anzellini, S. & Errandonea, D. Palladium at high pressure and high temperature: A combined experimental and theoretical study. *Journal of Applied Physics* **135**, 075103, 10.1063/5.0179469 (2024). https://pubs.aip.org/aip/jap/article-pdf/doi/10.1063/5.0179469/19801622/075103_1_5.0179469.pdf.

[6] Anzellini, S. et al. In situ characterization of the high pressure – high temperature melting curve of platinum. *Scientific Reports* **9**, 10.1038/s41598-019-49676-y (2019). 10.1038/s41598-019-49676-y PMC6736956 31506567

[7] Anzellini, S. et al. Thermal equation of state of ruthenium characterized by resistively heated diamond anvil cell. *Scientific Reports* **9**, 1–11. 10.1038/s41598-019-51037-8 (2019). 31595017 10.1038/s41598-019-51037-8 PMC6783540

[8] Wang, H. & Abruña, H. D. Rh and Rh alloy nanoparticles as highly active H_2_ oxidation catalysts for alkaline fuel cells. *ACS Catalysis* **9**, 5057–5062. 10.1021/acscatal.9b00906 (2019).

[9] Khishchenko, K. V. Equation of state for rhodium at high pressures. *Journal of Physics: Conference Series* **2057**, 012118. 10.1088/1742-6596/2057/1/012118 (2021).

[10] Walsh, J. M., Rice, M. H., McQueen, R. G. & Yarger, F. L. Shock-wave compressions of twenty-seven metals. equations of state of metals. *Phys. Rev.* **108**, 196–216. 10.1103/PhysRev.108.196 (1957).

[11] Young, D. A., Cynn, H., Söerlind, P. & Landa, A. Zero-Kelvin Compression Isotherms of the Elements 1 Z 92 to 100 GPa. *Journal of Physical and Chemical Reference Data* **45**, 043101, 10.1063/1.4963086 (2016). https://pubs.aip.org/aip/jpr/article-pdf/doi/10.1063/1.4963086/19747905/043101_1_online.pdf.

[12] Yusenko, K. V. et al. Equations of state of rhodium, iridium and their alloys up to 70 GPa. *Journal of Alloys and Compounds* **788**, 212–218. 10.1016/j.jallcom.2019.02.206 (2019).

[13] Frost, M., Smith, D., McBride, E. E., Smith, J. S. & Glenzer, S. H. The equations of state of statically compressed palladium and rhodium. *Journal of Applied Physics* **134**, 035901, 10.1063/5.0161038 (2023). https://pubs.aip.org/aip/jap/article-pdf/doi/10.1063/5.0161038/18050562/035901_1_5.0161038.pdf.

[14] McHardy, J. D. et al. Thermal equation of state of rhodium to 191 GPa and 2700 K using double-sided flash laser heating in a diamond anvil cell. *Phys. Rev. B* **109**, 094113. 10.1103/PhysRevB.109.094113 (2024).

[15] Storm, C. V. et al. The stress state in bismuth to 298 GPa and its use as a pressure transmitting medium and pressure marker at multi-megabar pressures. *Journal of Applied Physics* **133**, 245904 (2023).

[16] Kumar, P., Bhatt, N., Vyas, P. & Gohel, V. Thermodynamic properties of rhodium at high temperature and pressure by using mean field potential approach. *Eur. Phys. J. B* **89**, 219 (2016).

[17] A. E. Gheribi, J. R., & Roussel, J.-M. Phenomenological hugoniot curves for transition metals up to 1 TPa. *J. Phys. Condens. Matter* **19**, 476218 (2007).

[18] Cazorla, C., M. G. & Alfe, D. Zero-temperature generalized phase diagram of the 4d transition metals under pressure. *Phys. Rev. B* **77**, 224103 (2008).

[19] Cazorla, C., M. G. & Alfe, D. A simple tight-binding model for the study of 4d transition metals under pressure. *Comput. Mater. Sci.* **50**, 2732–2735 (2011).

[20] Huijina, T. et al. First-principles study on the lattice stability of elemental Co, Rh, and Ir in the VIIIB group. *Rare metals* **28**, 212–220 (2009).

[21] Smirnov, N. A. Ab initio calculations of structural stability, thermodynamic and elastic properties of Ni, Pd, Rh, and Ir at high pressures. *Journal of Applied Physics* **134**, 025901, 10.1063/5.0158737 (2023). https://pubs.aip.org/aip/jap/article-pdf/doi/10.1063/5.0158737/18040424/025901_1_5.0158737.pdf.

[22] Strong, H. M. & Bundy, F. P. Fusion curves of four group VIII metals to 100 000 atmospheres. *Phys. Rev.* **115**, 278–284. 10.1103/PhysRev.115.278 (1959).

[23] Ciftci, Y. O., Colakoglu, K., Ozgen, S. & Kazanc, S. The calculation of some thermoelastic properties and pressure-temperature (P-T) diagrams of Rh and Sr using molecular dynamics simulation. *J. Phys.: Condens. Matter* **19**, 326204 (2007).

[24] Swift, D. C. et al. Equations of state for ruthenium and rhodium (2019). arXiv:1909.05391.

[25] Anzellini, S. & Boccato, S. A Practical Review of the Laser-Heated Diamond Anvil Cell for University Laboratories and Synchrotron Applications. *Crystals* **10**, 459 (2020).

[26] Louvel, M. et al. The HXD95: a modified Bassett-type hydrothermal diamond-anvil cell for in situ XRD experiment up to 5 GPa and 1300 K. *Journal of Synchrotron Radiation* **27**, 529 (2020). 32153294 10.1107/S1600577519016801 PMC7064104

[27] Anzellini, S., Dewaele, A., Occelli, F., Loubeyre, P. & Mezouar, M. Equation of state of rhenium and application for ultra high pressure calibration. *Journal of Applied Physics* **115**, 043511, 10.1063/1.4863300 (2014). https://pubs.aip.org/aip/jap/article-pdf/doi/10.1063/1.4863300/15127717/043511_1_online.pdf.

[28] Takemura, K. & Dewaele, A. Isothermal equation of state for gold with a He-pressure medium. *Phys. Rev. B* **78**, 104119. 10.1103/PhysRevB.78.104119 (2008).

[29] Takemura, K. Evaluation of the hydrostaticity of a helium-pressure medium with powder x-ray diffraction techniques. *Journal of Applied Physics* **89**, 662–668, 10.1063/1.1328410 (2001). https://pubs.aip.org/aip/jap/article-pdf/89/1/662/19277080/662_1_online.pdf.

[30] Storm, C. V. et al. The stress state in bismuth to 298 GPa and its use as a pressure transmitting medium and pressure marker at multi-megabar pressures. *Journal of Applied Physics* **133**, 245904, 10.1063/5.0150419 (2023). https://pubs.aip.org/aip/jap/article-pdf/doi/10.1063/5.0150419/18025680/245904_1_5.0150419.pdf.

[31] Dewaele, A., Loubeyre, P. & Mezouar, M. Equations of state of six metals above 94 GPa. *Phys. Rev. B* **70**, 094112. 10.1103/PhysRevB.70.094112 (2004).

[32] Dewaele, A., Torrent, M., Loubeyre, P. & Mezouar, M. Compression curves of transition metals in the mbar range: Experiments and projector augmented-wave calculations. *Phys. Rev. B* **78**, 104102. 10.1103/PhysRevB.78.104102 (2008).

[33] Maurer, D., Heichele, R., Lingg, N., Muller, V. & Rieder, K. Elastic Properties of Purified Single-Crystalline Rhodium. *Phys. Stat. Sol.* **160**, 403 (1997).

[34] Shao, Z. et al. First-principles investigation of rhodium hydrides under high pressure. *Phys. Rev. B* **104**, 054110. 10.1103/PhysRevB.104.054110 (2021).

[35] Vinet, P., Ferrante, J., Smith, J. R. & Rose, J. H. A universal equation of state for solids. *Journal of Physics C: Solid State Physics* **19**, L467. 10.1088/0022-3719/19/20/001 (1986).

[36] Angel, R. J., Alvaro, M. & González-Platas, J. Eosfit7c and a fortran module (library) for equation of state calculations. *Zeitschrift für Kristallographie - Crystalline Materials* **229**, 405–419 (2014).

[37] Eichler, A., Bohnen, K.-P., Reichardt, W. & Hafner, J. Phonon dispersion relation in rhodium: Ab initio calculations and neutron- scattering investigations. *Phys. Rev. B* **57**, 324–333. 10.1103/PhysRevB.57.324 (1998).

[38] Walker, et al. Elastic moduli of rhodium: Correct prediction by a new theoretical method. *Phys. Rev. B* **24**, 2254–2256. 10.1103/PhysRevB.24.2254 (1981).

[39] Simmons, G. & Wang, H. F. Single crystal elastic constants and calculated aggregate properties. a handbook (1971).

[40] Gao, J., Liu, Q.-J. & Tang, B. Elastic stability criteria of seven crystal systems and their application under pressure: Taking carbon as an example. *Journal of Applied Physics* **133**, 135901, 10.1063/5.0139232 (2023). https://pubs.aip.org/aip/jap/article-pdf/doi/10.1063/5.0139232/16824213/135901_1_5.0139232.pdf.

[41] Wang, J., Li, J., Yip, S., Wolf, D. & Phillpot, S. Unifying two criteria of born: Elastic instability and melting of homogeneous crystals. *Physica A: Statistical Mechanics and its Applications* **240**, 396–403, 10.1016/S0378-4371(97)00161-1 (1997). Proceedings of the Euroconference on the microscopic approach to complexity in non-equilibrium molecular simulations.

[42] Slaughter, W. *The Linearized Theory of Elasticity* (Birkhäuser Boston, 2002).

[43] Pugh, S. X. C. I. I. Relations between the elastic moduli and the plastic properties of polycrystalline pure metals. *The London, Edinburgh, and Dublin Philosophical Magazine and Journal of Science* **45**, 823–843. 10.1080/14786440808520496 (1954).

[44] Dewaele, A. et al. High-pressure–high-temperature equation of state of KCl and KBr. *Physical Review B* **85**, 214105 (2012).

[45] Mao, H. & Bell, P. High-Pressure Physics: The 1-Megabar Mark on the Ruby R1 Static Pressure Scale. *Science* **191**, 851 (1976). 17730998 10.1126/science.191.4229.851

[46] Rosa, A. et al. Amorphous boron composite gaskets for in situ high-pressure and high-temperature studies. *High Pressure Research* **36**, 564 (2016).

[47] Garbarino, G. et al. Extreme conditions x-ray diffraction and imaging beamline id15b on the esrf extremely brilliant source. *High Pressure Research* **44**, 199–216. 10.1080/08957959.2024.2379294 (2024).

[48] Prescher, C. & Prakapenka, V. B. DIOPTAS: a program for reduction of two-dimensional X-ray diffraction data and data exploration. *High Pressure Research* **35**, 223–230 (2015).

[49] Coelho, A. A. TOPAS and TOPAS-Academic: an optimization program integrating computer algebra and crystallographic objects written in C++. *Journal of Applied Crystallography* **51**, 210–218. 10.1107/S1600576718000183 (2018).

[50] Kresse, G. & Joubert, D. From ultrasoft pseudopotentials to the projector augmented-wave method. *Phys. Rev. B* **59**, 1758–1775. 10.1103/PhysRevB.59.1758 (1999).

[51] Blöchl, P. E. Projector augmented-wave method. *Phys. Rev. B* **50**, 17953–17979. 10.1103/PhysRevB.50.17953 (1994). 10.1103/physrevb.50.17953 9976227

[52] Perdew, J. P. et al. Restoring the density-gradient expansion for exchange in solids and surfaces. *Phys. Rev. Lett.* **100**, 136406. 10.1103/PhysRevLett.100.136406 (2008). 18517979 10.1103/PhysRevLett.100.136406

[53] Cazorla, C., Errandonea, D. & Sola, E. High-pressure phases, vibrational properties, and electronic structure of Ne(He)_2_ and Ar(He)_2_: A first-principles study. *Phys. Rev. B* **80**, 064105. 10.1103/PhysRevB.80.064105 (2009).

[54] Cazorla, C. & Boronat, J. Simulation and understanding of atomic and molecular quantum crystals. *Rev. Mod. Phys.* **89**, 035003. 10.1103/RevModPhys.89.035003 (2017).

[55] Togo, A., Chaput, L., Tadano, T. & Tanaka, I. Implementation strategies in phonopy and phono3py. *J. Phys. Condens. Matter* **35**, 353001. 10.1088/1361-648X/acd831 (2023). 10.1088/1361-648X/acd831 37220761

