# Supplementary material for: Thermal equation of state of rhodium characterized by XRD in a resistively heated diamond anvil cell
Source: Sci Rep. 2024 Nov 4;14:26634. doi: 10.1038/s41598-024-78006-0 (PMC11535440; doi:10.1038/s41598-024-78006-0)
Supplement: Supplementary file 1 — Supplementary Information. [file 41598_2024_78006_MOESM1_ESM.pdf]

Supplementary Material: Thermal equation of state of rhodium characterized by resistively heated diamond anvil cell

Jose Luis Rodrigo Ramon[1], Simone Anzellini[1,*], Claudio Cazorla[3], Pablo Botella[1], Aser Garcia-Beamud[1], Josu Sanchez-Martin[1,2], Gaston Garbarino[2], Angelika Rosa[2], Samuel Gallego Parra[2], and Daniel Errandonea[1]

[1]*Department of Applied Physics - Institute of Materials Science, Matter at High Pressure (MALTA) Consolider Team, University of Valencia, C/Dr. Moliner 50, Burjassot, 46100 Valencia, Spain*
[2]*Department of Physics, Universitat Politècnica de Catalunya, Campus Nord B4-B5, Barcelona 08034, Spain*
[3]*European Synchrotron Radiation Facility, 71 Avenue des Martyrs, Grenoble, CS 40220, 38043, France*
[*]*simone2.anzellini@uv.es*

September 2024

0.1 HIGH TEMPERATURE RESISTIVE HEATING OF RH

T(K)	a_{KCl} (Å)	Volume KCl (Å^3)	a_{Rh} (Å)	Volume Rh (Å^3)	P(GPa)
300	3.658	48.96	3.793	54.57	2.53
300	3.658	48.95	3.793	54.58	2.53
300	3.657	48.92	3.793	54.58	2.55
300	3.656	48.90	3.793	54.58	2.57
300	3.656	48.89	3.793	54.58	2.57
300	3.656	48.87	3.793	54.60	2.59
300	3.656	48.87	3.793	54.57	2.58
300	3.655	48.85	3.793	54.60	2.59
300	3.656	48.86	3.793	54.57	2.59
300	3.655	48.85	3.793	54.60	2.60
300	3.655	48.86	3.793	54.57	2.59
300	3.655	48.83	3.793	54.60	2.61
300	3.655	48.85	3.793	54.57	2.60
300	3.654	48.82	3.793	54.59	2.62
300	3.655	48.84	3.792	54.56	2.60
300	3.655	48.86	3.793	54.60	2.59
300	3.656	48.89	3.793	54.57	2.57
300	3.655	48.86	3.793	54.59	2.59
300	3.656	48.89	3.793	54.57	2.57
300	3.655	48.86	3.793	54.59	2.59
300	3.656	48.89	3.793	54.57	2.57
300	3.653	48.78	3.792	54.56	2.64
300	3.653	48.78	3.793	54.57	2.64
300	3.646	48.48	3.793	54.57	2.84
300	3.646	48.47	3.792	54.55	2.84
300	3.634	47.99	3.792	54.55	3.17
300	3.635	48.03	3.791	54.48	3.14
300	3.624	47.60	3.790	54.47	3.45
300	3.623	47.56	3.789	54.42	3.48
300	3.610	47.07	3.788	54.37	3.86
300	3.612	47.14	3.787	54.31	3.80
300	3.588	46.20	3.784	54.21	4.58
300	3.586	46.12	3.784	54.21	4.65
300	3.580	45.91	3.783	54.15	4.84
300	3.576	45.75	3.784	54.18	4.99
300	3.572	45.58	3.781	54.05	5.14
300	3.570	45.50	3.782	54.12	5.22
300	3.561	45.17	3.779	53.99	5.55
300	3.561	45.18	3.781	54.06	5.54
300	3.551	44.80	3.777	53.91	5.92
300	3.551	44.80	3.780	54.02	5.92
300	3.542	44.45	3.776	53.84	6.29
300	3.540	44.38	3.778	53.93	6.37
300	3.520	43.64	3.773	53.72	7.23
300	3.516	43.50	3.775	53.79	7.39
500	3.458	41.36	3.763	53.31	10.53
500	3.466	41.64	3.763	53.31	10.10
500	3.465	41.62	3.766	53.42	10.13
600	3.355	37.77	3.757	53.04	18.22
600	3.386	38.84	3.748	52.66	15.70
600	3.384	38.76	3.746	52.60	15.89
600	3.376	38.47	3.744	52.49	16.53

600	3.371	38.33	3.743	52.44	16.86
600	3.366	38.13	3.741	52.37	17.33
600	3.357	37.84	3.739	52.27	18.04
600	3.346	37.48	3.735	52.13	18.96
600	3.339	37.24	3.733	52.04	19.60
600	3.331	36.97	3.731	51.94	20.34
600	3.320	36.59	3.726	51.74	21.43
600	3.312	36.34	3.723	51.61	22.19
600	3.289	35.60	3.717	51.38	24.53
600	3.280	35.31	3.713	51.22	25.51
600	3.271	35.00	3.708	51.00	26.63
600	3.266	34.83	3.705	50.88	27.23
600	3.230	33.71	3.692	50.33	31.73
600	3.226	33.57	3.691	50.30	32.32
600	3.226	33.59	3.690	50.26	32.26
600	3.223	33.49	3.689	50.21	32.69
600	3.216	33.27	3.685	50.07	33.70
600	3.213	33.18	3.684	50.02	34.11
800	3.109	30.06	3.637	48.11	52.64
800	3.112	30.14	3.637	48.14	52.07
800	3.115	30.25	3.639	48.20	51.35
800	3.116	30.28	3.639	48.21	51.13
800	3.122	30.44	3.640	48.25	49.99
800	3.126	30.56	3.645	48.44	49.18
800	3.125	30.53	3.645	48.44	49.38
800	3.125	30.52	3.645	48.45	49.48
800	3.125	30.53	3.645	48.46	49.39
800	3.128	30.62	3.646	48.50	48.82
800	3.131	30.69	3.647	48.53	48.33
800	3.131	30.72	3.648	48.55	48.15
800	3.132	30.73	3.648	48.56	48.09
800	3.131	30.72	3.648	48.57	48.17
800	3.132	30.73	3.648	48.58	48.09
800	3.133	30.75	3.649	48.58	47.96
800	3.133	30.76	3.649	48.59	47.88
800	3.133	30.77	3.649	48.60	47.83
800	3.138	30.90	3.649	48.61	46.99
800	3.125	30.53	3.650	48.62	49.42
800	3.125	30.52	3.650	48.63	49.49
800	3.135	30.83	3.650	48.64	47.43
800	3.133	30.77	3.650	48.65	47.81
800	3.139	30.95	3.651	48.66	46.66
800	3.145	31.13	3.653	48.78	45.53
800	3.149	31.23	3.654	48.81	44.94
800	3.145	31.13	3.655	48.84	45.56
800	3.149	31.23	3.657	48.91	44.90
800	3.155	31.42	3.658	48.96	43.75
800	3.123	30.47	3.644	48.41	49.77
800	3.191	32.51	3.677	49.74	37.81
800	3.192	32.53	3.678	49.76	37.70
800	3.192	32.55	3.679	49.79	37.60
800	3.193	32.57	3.680	49.83	37.50
800	3.193	32.57	3.681	49.88	37.47
800	3.196	32.64	3.682	49.93	37.13
800	3.197	32.70	3.684	50.01	36.85

800	3.203	32.87	3.688	50.17	36.03
800	3.207	32.99	3.691	50.30	35.42
800	3.147	31.18	3.658	48.95	45.23
800	3.152	31.31	3.660	49.06	44.41
800	3.161	31.59	3.664	49.19	42.81
800	3.162	31.63	3.664	49.20	42.57
800	3.163	31.66	3.664	49.19	42.37

Table S1: The P-V -T data for Rh and KCl measured during the resistive heating cycles. The sample pressure was determined using the KCl-B2 equations of state.

0.2 LOCAL STRESS

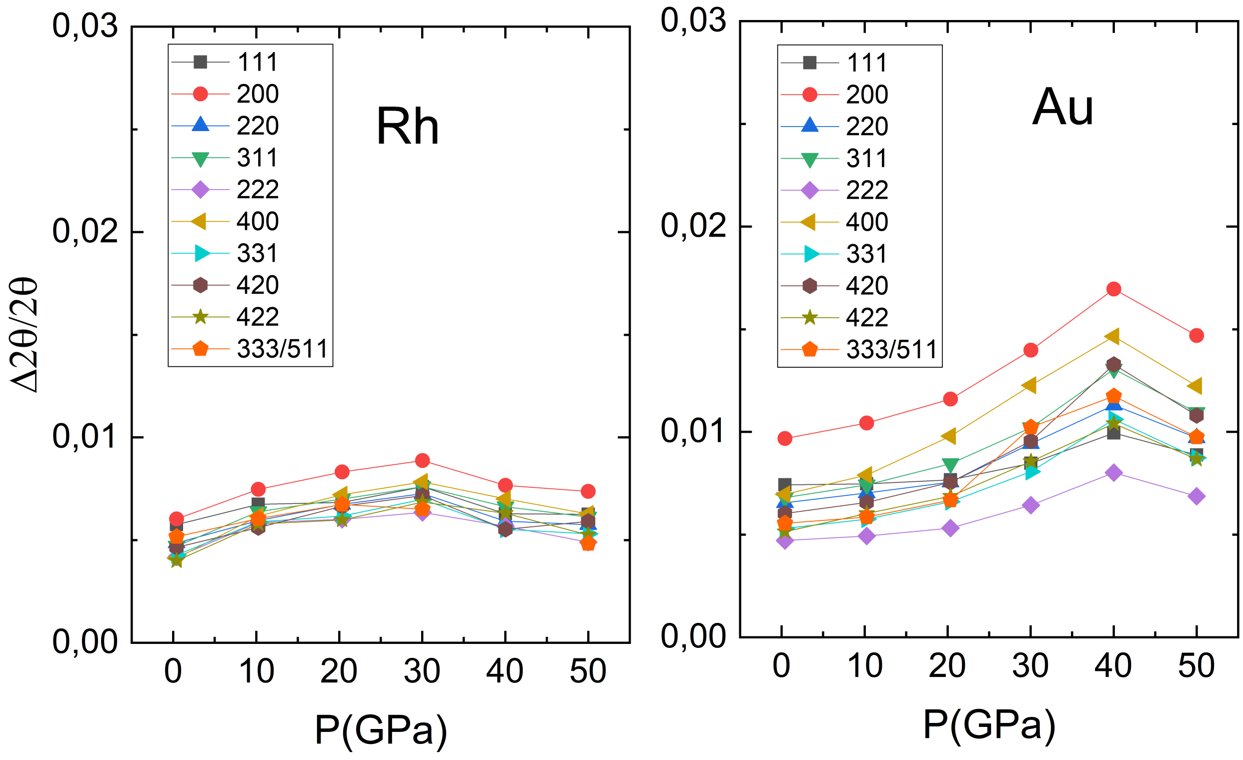

Figure S1: Evolution with the pressure of the FWHM ($\Delta 2\theta$) over 2θ.

In order to establish the Equation of State (EOS), precise and accurate knowledge of both sample pressure and volume is necessary. While attaining purely hydrostatic conditions in the sample chamber is preferred, it is worth noting that even helium solidifies above approximately 12 GPa at room temperature. Consequently, the stress condition beyond this pressure threshold may deviate from hydrostatic, necessitating a meticulous assessment of stress states in each experiment In addition, even at lower pressure, the stress on the sample can become non-hydrostatic if it bridges the anvils due to excessive thinning of the gasket or due to a large initial thickness of the sample. We can evaluate the stress state of the sample from the shift and broadening of x-ray diffraction peaks. The d spacings, if normalized to the values at atmospheric pressure d_0, should show the same compression under hydrostatic pressure irrespective of hkl indices. On the other hand, under non-hydrostatic conditions, the normalized d spacings become different for each hkl index due to the elastic anisotropy.

With the 2θ values obtained for each reflection, we can calculate each interplanar distance and compare it with the values obtained from the unit cell parameter. We will refer as d_m to the interplanar distances obtained from the 2θ values and d_c the ones calculated from the unit cell parameter obtained with Pawley fitting.

A preliminary study of the stress for the sample and pressure gauge has been made and it is represented in figures S1 and S2. From this study, we can infer that Rh is less affected by the non-hydrostatic effects than Au. Moreover, the effects begin to become visible starting from 25-30 GPa, reaching their peak at the moment of diamond rupture

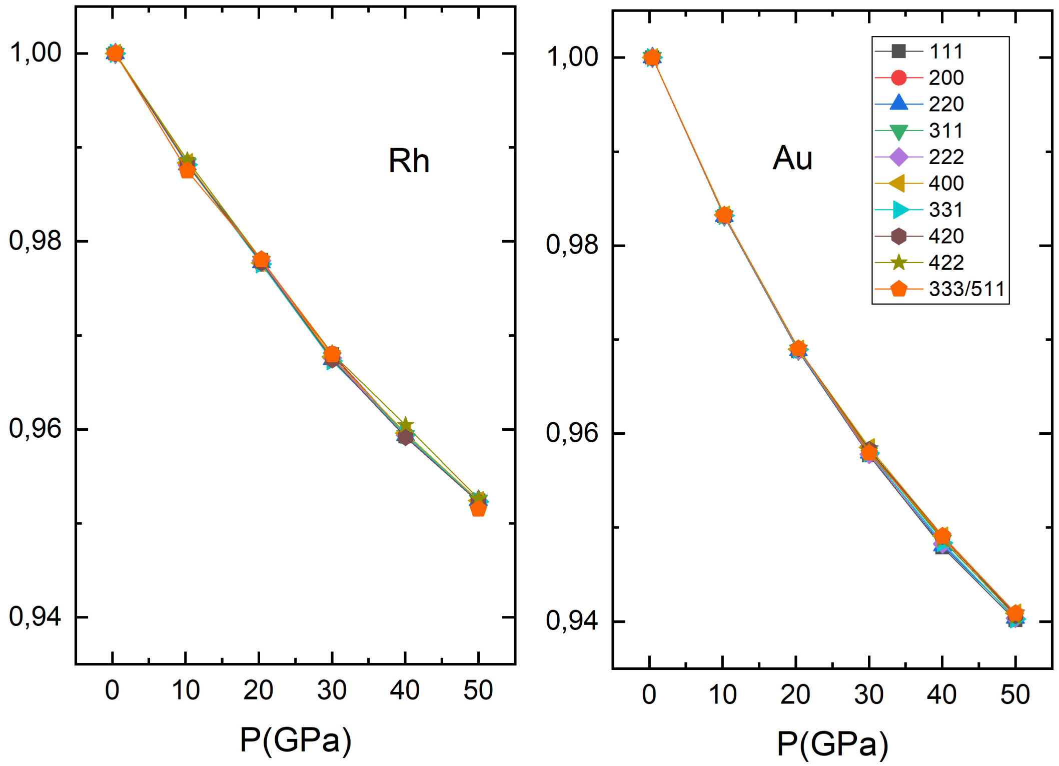

Figure S2: Normalized d-spacings of Rh and Au as a function of pressure. Figure shows the details of the splitting of d/d_0 depending on hkl indices.

0.3 ELASTIC CONSTANTS OF RH TO 140 GPa

P(GPa)	C_{11} (GPa)	C_{12} (GPa)	C_{44} (GPa)	P(GPa)	C_{11} (GPa)	C_{12} (GPa)	C_{44} (GPa)
0	466,894	207,13	208,217	68,80302	813,946	411,229	370,464
0,68803	471,322	209,646	210,289	69,49105	816,941	413,054	371,899
1,37606	475,747	212,159	212,36	70,17908	819,936	414,883	373,332
2,06409	480,165	214,668	214,428	70,86711	822,929	416,718	374,761
2,75212	484,574	217,17	216,49	71,55514	825,921	418,557	376,187
3,44015	488,97	219,663	218,547	72,24317	828,911	420,4	377,61
4,12818	493,351	222,144	220,595	72,9312	831,899	422,247	379,03
4,81621	497,712	224,611	222,634	73,61923	834,886	424,098	380,447
5,50424	502,051	227,061	224,662	74,30726	837,87	425,951	381,861
6,19227	506,364	229,493	226,676	74,99529	840,852	427,808	383,271
6,8803	510,649	231,904	228,677	75,68332	843,832	429,668	384,678
7,56833	514,902	234,292	230,661	76,37135	846,809	431,53	386,081
8,25636	519,12	236,653	232,628	77,05938	849,783	433,395	387,482
8,94439	523,3	238,987	234,575	77,74741	852,755	435,261	388,879
9,63242	527,438	241,29	236,502	78,43544	855,723	437,13	390,273
10,32045	531,532	243,561	238,406	79,12347	858,688	438,999	391,663
11,00848	535,577	245,796	240,286	79,8115	861,649	440,87	393,05
11,69651	539,573	247,995	242,141	80,49953	864,607	442,742	394,434
12,38454	543,521	250,159	243,971	81,18756	867,56	444,614	395,814
13,07257	547,421	252,291	245,778	81,87559	870,51	446,487	397,191

13,7606	551,278	254,393	247,563	82,56362	873,456	448,36	398,565
14,44863	555,093	256,467	249,326	83,25165	876,397	450,233	399,935
15,82469	562,604	260,544	252,794	84,62771	882,266	453,976	402,664
16,51272	566,306	262,551	254,5	85,31574	885,193	455,847	404,024
17,20075	569,974	264,541	256,189	86,00377	888,116	457,716	405,38
17,88878	573,611	266,515	257,862	86,6918	891,032	459,584	406,732
18,57681	577,218	268,477	259,52	87,37983	893,944	461,45	408,081
19,26484	580,799	270,429	261,164	88,06786	896,85	463,314	409,427
19,95287	584,355	272,372	262,796	88,75589	899,75	465,175	410,768
20,6409	587,889	274,311	264,415	89,44392	902,645	467,034	412,106
21,32893	591,402	276,247	266,025	90,13195	905,533	468,89	413,441
22,01696	594,898	278,182	267,624	90,81998	908,415	470,743	414,772
22,70499	598,377	280,119	269,215	91,50801	911,29	472,593	416,099
23,39303	601,842	282,061	270,799	92,19604	914,159	474,439	417,423
24,08106	605,295	284,01	272,376	92,88407	917,021	476,281	418,742
24,76909	608,739	285,968	273,948	93,5721	919,877	478,118	420,058
25,45712	612,176	287,939	275,516	94,26013	922,725	479,952	421,371
26,14515	615,608	289,923	277,081	94,94816	925,565	481,78	422,679
26,83318	619,036	291,924	278,643	95,63619	928,399	483,603	423,984
27,52121	622,461	293,941	280,203	96,32422	931,224	485,422	425,285
28,20924	625,883	295,972	281,761	97,01225	934,042	487,234	426,582
28,89727	629,301	298,017	283,318	97,70028	936,852	489,041	427,876
29,5853	632,714	300,075	284,872	98,38831	939,654	490,841	429,165
30,27333	636,124	302,143	286,425	99,07634	942,447	492,636	430,451
30,96136	639,528	304,222	287,975	99,76437	945,232	494,423	431,733
31,64939	642,928	306,309	289,524	100,4524	948,008	496,204	433,01
32,33742	646,322	308,404	291,071	101,14043	950,776	497,978	434,285
33,02545	649,71	310,506	292,615	101,82846	953,536	499,746	435,555
34,40151	656,467	314,723	295,7	103,20452	959,032	503,264	438,085
35,08954	659,836	316,837	297,239	103,89255	961,768	505,013	439,345
35,77757	663,197	318,953	298,777	104,58058	964,497	506,756	440,601
36,4656	666,551	321,069	300,313	105,26861	967,218	508,494	441,854
37,15363	669,897	323,184	301,847	105,95664	969,932	510,226	443,104
37,84166	673,235	325,298	303,38	106,64467	972,639	511,953	444,35
38,52969	676,565	327,408	304,911	107,3327	975,34	513,674	445,594
39,21772	679,886	329,514	306,44	108,02073	978,033	515,389	446,834
39,90575	683,197	331,615	307,968	108,70876	980,72	517,1	448,071
40,59378	686,499	333,71	309,494	109,39679	983,4	518,805	449,306
41,28181	689,792	335,796	311,018	110,08482	986,074	520,506	450,537
41,96984	693,074	337,874	312,541	110,77285	988,742	522,201	451,766
42,65787	696,345	339,941	314,062	111,46088	991,404	523,892	452,992
43,3459	699,606	341,997	315,582	112,14891	994,06	525,578	454,215
44,03393	702,855	344,041	317,1	112,83694	996,71	527,26	455,436
44,72196	706,093	346,071	318,617	113,52497	999,355	528,938	456,654
45,40999	709,32	348,086	320,132	114,21301	1001,99	530,611	457,87
46,09802	712,534	350,086	321,646	114,90104	1004,63	532,28	459,083
46,78605	715,736	352,071	323,158	115,58907	1007,26	533,945	460,294
47,47408	718,927	354,041	324,668	116,2771	1009,88	535,606	461,503
48,16211	722,107	355,997	326,177	116,96513	1012,5	537,264	462,71
48,85014	725,277	357,94	327,685	117,65316	1015,12	538,918	463,915
49,53817	728,436	359,871	329,19	118,34119	1017,73	540,568	465,117
50,2262	731,584	361,789	330,694	119,02922	1020,33	542,215	466,318
50,91423	734,723	363,696	332,196	119,71725	1022,93	543,859	467,517
51,60226	737,853	365,593	333,697	120,40528	1025,53	545,5	468,714
52,29029	740,973	367,478	335,195	121,09331	1028,13	547,137	469,909

52,97832	744,084	369,355	336,691	121,78134	1030,72	548,772	471,102
53,66635	747,187	371,222	338,186	122,46937	1033,3	550,404	472,294
54,35438	750,282	373,081	339,678	123,1574	1035,89	552,033	473,485
55,04241	753,369	374,932	341,168	123,84543	1038,47	553,66	474,674
55,73044	756,448	376,776	342,657	124,53346	1041,04	555,285	475,861
56,41847	759,52	378,613	344,143	125,22149	1043,62	556,907	477,048
57,1065	762,585	380,444	345,626	125,90952	1046,19	558,527	478,233
57,79453	765,644	382,269	347,108	126,59755	1048,76	560,144	479,416
58,48256	768,696	384,09	348,587	127,28558	1051,33	561,76	480,599
59,17059	771,743	385,907	350,064	127,97361	1053,89	563,375	481,781
59,85862	774,783	387,719	351,538	128,66164	1056,45	564,987	482,961
60,54665	777,819	389,529	353,01	129,34967	1059,01	566,598	484,141
61,23468	780,849	391,337	354,479	130,0377	1061,57	568,208	485,32
61,92271	783,875	393,142	355,946	130,72573	1064,13	569,816	486,499
62,61074	786,896	394,947	357,41	131,41376	1066,68	571,423	487,676
63,29877	789,914	396,75	358,872	132,10179	1069,24	573,029	488,853
63,9868	792,927	398,554	360,331	132,78982	1071,79	574,634	490,029
64,67483	795,938	400,358	361,787	133,47785	1074,34	576,238	491,205
65,36286	798,945	402,164	363,24	134,16588	1076,89	577,841	492,381
66,05089	801,949	403,971	364,691	134,85391	1079,44	579,444	493,556
66,73892	804,951	405,78	366,139	135,54194	1081,99	581,047	494,731
67,42695	807,951	407,593	367,583	136,22997	1084,54	582,649	495,906
68,11498	810,949	409,409	369,025	136,918	1087,09	584,25	497,08

Table S2: Calculated elastic constants (C_{11}, C_{12} and C_{44}) of Rhodium (Rh) as a function of pressure, up to 136 GPa, obtained through density functional theory (DFT) calculations.

0.4 CALCULATED EOS ISOTHERMS FOR RHODIUM

	V (Å^3)			
**P(GPa)**	**T= 300 K**	**T= 500 K**	**T= 600 K**	**T= 800 K**
0,36123	54,971	55,37	55,584	56,035
0,98144	54,837	55,229	55,44	55,883
1,6017	54,705	55,091	55,298	55,734
2,2219	54,576	54,956	55,159	55,587
2,8421	54,448	54,822	55,022	55,443
3,4623	54,323	54,691	54,888	55,302
4,0825	54,199	54,562	54,755	55,163
4,7027	54,077	54,434	54,625	55,026
5,323	53,957	54,309	54,497	54,891
5,9432	53,838	54,185	54,37	54,759
6,5634	53,722	54,064	54,246	54,628
7,1836	53,606	53,944	54,123	54,5
7,8038	53,493	53,825	54,002	54,373
8,424	53,381	53,709	53,883	54,249
9,0443	53,27	53,594	53,766	54,126
9,6645	53,161	53,48	53,65	54,005
10,285	53,053	53,368	53,536	53,886
10,905	52,947	53,258	53,423	53,769
11,525	52,842	53,149	53,312	53,653
12,145	52,738	53,041	53,202	53,539
12,766	52,636	52,935	53,094	53,426
13,386	52,534	52,83	52,987	53,315
14,006	52,434	52,726	52,882	53,205
14,626	52,335	52,624	52,777	53,097
15,246	52,238	52,523	52,674	52,99
15,867	52,141	52,423	52,573	52,884
16,487	52,046	52,324	52,472	52,78
17,107	51,951	52,227	52,373	52,677
17,727	51,858	52,13	52,275	52,575
18,348	51,765	52,035	52,178	52,475
18,968	51,674	51,941	52,082	52,375
19,588	51,584	51,847	51,987	52,277
20,208	51,494	51,755	51,893	52,18
20,828	51,406	51,664	51,8	52,084
21,449	51,318	51,574	51,709	51,989
22,069	51,231	51,484	51,618	51,895
22,689	51,146	51,396	51,528	51,803
23,309	51,061	51,308	51,439	51,711
23,929	50,977	51,222	51,351	51,62
24,55	50,893	51,136	51,264	51,53
25,17	50,811	51,051	51,178	51,441
25,79	50,729	50,967	51,093	51,353
26,41	50,648	50,884	51,009	51,266
27,031	50,568	50,802	50,925	51,18
27,651	50,489	50,72	50,842	51,095
28,271	50,41	50,639	50,76	51,011
28,891	50,332	50,559	50,679	50,927
29,511	50,255	50,48	50,599	50,844
30,132	50,179	50,402	50,519	50,762

30,752	50,103	50,324	50,44	50,681
31,372	50,028	50,247	50,362	50,601
31,992	49,953	50,17	50,285	50,521
32,612	49,879	50,094	50,208	50,442
33,233	49,806	50,019	50,132	50,364
33,853	49,734	49,945	50,056	50,286
34,473	49,662	49,871	49,982	50,21
35,093	49,59	49,798	49,908	50,134
35,714	49,52	49,726	49,834	50,058
36,334	49,45	49,654	49,761	49,983
36,954	49,38	49,582	49,689	49,909
37,574	49,311	49,512	49,618	49,836
38,194	49,242	49,442	49,547	49,763
38,815	49,174	49,372	49,476	49,691
39,435	49,107	49,303	49,406	49,619
40,055	49,04	49,235	49,337	49,548
40,675	48,974	49,167	49,268	49,478
41,296	48,908	49,1	49,2	49,408
41,916	48,843	49,033	49,133	49,339
42,536	48,778	48,967	49,066	49,27
43,156	48,714	48,901	48,999	49,202
43,776	48,65	48,836	48,933	49,134
44,397	48,586	48,771	48,868	49,067
45,017	48,524	48,707	48,803	49,001
45,637	48,461	48,643	48,738	48,935
46,257	48,399	48,579	48,674	48,869
46,877	48,338	48,517	48,611	48,804
47,498	48,277	48,454	48,548	48,74
48,118	48,216	48,392	48,485	48,676
48,738	48,156	48,331	48,423	48,612
49,358	48,096	48,27	48,361	48,549
49,979	48,037	48,209	48,3	48,486
50,599	47,978	48,149	48,239	48,424
51,219	47,919	48,089	48,179	48,363
51,839	47,861	48,03	48,119	48,301
52,459	47,803	47,971	48,059	48,24
53,08	47,746	47,913	48	48,18
53,7	47,689	47,854	47,941	48,12
54,32	47,632	47,797	47,883	48,061
54,94	47,576	47,739	47,825	48,001
55,56	47,52	47,682	47,768	47,943
56,181	47,465	47,626	47,71	47,884
56,801	47,41	47,57	47,654	47,827
57,421	47,355	47,514	47,597	47,769
58,041	47,3	47,458	47,541	47,712

Table S3: Calculated isotherms of Rhodium at 300 K, 500 K, 600 K and 800 K using the equation of state (EOS) fit to experimental data with V_0 =55.046(16) Å^3, K_0 = 251(3) GPa, K_0' = 5.7(2) and α_0 =3.36(7)x10^{-5}K^{-1}.

0.5 SIMULATED DFT ISOTHERMS FOR RHODIUM

	P(GPa)			
V(Å^3)	T=300 K	T=500 K	T=600 K	T=800 K
55,28056	-1,10763	0,29368	1,01033	2,44668
55,13026	-0,39038	1,00826	1,72375	3,15779
54,97996	0,33895	1,73488	2,44919	3,88089
54,82966	1,08056	2,47373	3,18684	4,61617
54,67936	1,83463	3,22501	3,9369	5,36381
54,52906	2,60137	3,9889	4,69957	6,12402
54,37876	3,38096	4,76562	5,47503	6,89698
54,22846	4,17361	5,55535	6,26349	7,68291
54,07816	4,97952	6,35831	7,06515	8,48201
53,92786	5,79891	7,17469	7,88023	9,29448
53,77755	6,63199	8,00473	8,70894	10,12054
53,62725	7,47896	8,84862	9,55148	10,96041
53,47695	8,34005	9,70659	10,40809	11,81429
53,32665	9,21549	10,57887	11,27898	12,68242
53,17635	10,1055	11,46567	12,16437	13,56502
53,02605	11,0103	12,36723	13,06451	14,46232
52,87575	11,93014	13,28378	13,97962	15,37456
52,72545	12,86525	14,21557	14,90995	16,30198
52,57515	13,81588	15,16283	15,85573	17,24482
52,42485	14,78227	16,12581	16,81722	18,20332
52,27455	15,76468	17,10477	17,79466	19,17774
52,12425	16,76335	18,09995	18,78832	20,16833
51,97395	17,77855	19,11163	19,79844	21,17536
51,82365	18,81055	20,14006	20,8253	22,19909
51,67335	19,85961	21,18551	21,86917	23,23978
51,52305	20,92601	22,24826	22,93031	24,29772
51,37275	22,01002	23,32858	24,00901	25,37318
51,22244	23,11193	24,42677	25,10556	26,46645
51,07214	24,23203	25,5431	26,22024	27,57781
50,92184	25,37061	26,67788	27,35334	28,70757
50,77154	26,52797	27,8314	28,50516	29,85601
50,62124	27,70442	29,00396	29,67602	31,02345
50,47094	28,90026	30,19588	30,86622	32,21019
50,32064	30,11581	31,40748	32,07607	33,41656
50,17034	31,35138	32,63906	33,30589	34,64287
50,02004	32,60732	33,89097	34,55602	35,88945
49,86974	33,88394	35,16353	35,82679	37,15663
49,71944	35,18159	36,45707	37,11853	38,44476
49,56914	36,50061	37,77196	38,43159	39,75417
49,41884	37,84136	39,10853	39,76633	41,08523
49,26854	39,20418	40,46714	41,12309	42,43828
49,11824	40,58944	41,84817	42,50225	43,8137
48,96794	41,99752	43,25197	43,90417	45,21185
48,81763	43,42878	44,67892	45,32923	46,63311
48,66733	44,88362	46,12942	46,77782	48,07788
48,51703	46,36242	47,60385	48,25033	49,54653
48,36673	47,86558	49,10261	49,74715	51,03948
48,21643	49,3935	50,6261	51,26869	52,55712
48,06613	50,9466	52,17474	52,81538	54,09987

47,91583	52,5253	53,74895	54,38761	55,66816
47,76553	54,13003	55,34916	55,98584	57,26241
47,61523	55,76121	56,9758	57,61049	58,88307
47,46493	57,41931	58,62932	59,26201	60,53057
47,31463	59,10475	60,31017	60,94085	62,20537
47,16433	60,81802	62,01882	62,64747	63,90794
47,01403	62,55957	63,75573	64,38235	65,63874
46,86373	64,32989	65,52138	66,14597	67,39826
46,71343	66,12946	67,31626	67,93881	69,187
46,56313	67,95878	69,14088	69,76136	71,00543
46,41283	69,81835	70,99572	71,61415	72,85408
46,26252	71,70868	72,88132	73,49768	74,73347
46,11222	73,63031	74,79819	75,41249	76,64412
45,96192	75,58377	76,74688	77,3591	78,58657
45,81162	77,5696	78,72792	79,33807	80,56136
45,66132	79,58836	80,74188	81,34995	82,56907
45,51102	81,64061	82,78933	83,39532	84,61025
45,36072	83,72693	84,87084	85,47474	86,68549
45,21042	85,84791	86,98699	87,58882	88,79538
45,06012	88,00415	89,1384	89,73815	90,94053

Table S4: Calculated isotherms of Rhodium at 300 K, 500 K, 600 K, and 800 K obtained using density functional theory (DFT) calculations.

0.6 COVARIANCE MATRICES OF DIFFERENT EOS FITS

Form	$\mathbf{V_0}$ (**Å**^{3})	$\mathbf{K_0}$ **(GPa)**	$\mathbf{K'}$	$\sigma^2_{\mathbf{K_0K'}}$	$\sigma^2_{\mathbf{K_0V_0}}$	$\sigma^2_{\mathbf{K'V_0}}$
BM3	55.045(15)	252(3)	5.5(2)	-0.227163	-0.016100	0.0007465
Vinet	55.046(16)	251(3)	5.7(2)	-0,475808	-0.015837	0.0007090

Table S5: Covariances ($\sigma^2_{i,j}$)for the best-fitting parameters of the ambient isothermal compression data of Table 1. These uncertainities have been used to compute the 95% interval of confidence of the fitted EoS.
